# Vibronic Reorganization Suppresses Salinixanthin-to-Retinal Energy Transfer in the Freshwater Kin4B8 Xanthorhodopsin

**DOI:** 10.1021/acs.jpclett.6c00507

**Published:** 2026-04-07

**Authors:** Giacomo Salvadori, Piermarco Saraceno, Chris John, Lorenzo Cupellini, Laura Pedraza-González

**Affiliations:** † Institute for Computational Biomedicine (INM-9), Forschungszentrum Jülich, 52428 Jülich, Germany; ‡ Dipartimento di Chimica e Chimica Industriale, Università di Pisa, via G. Moruzzi 13, 56124 Pisa, Italy

## Abstract

Salinixanthin, a 4-keto xanthophyll, acts as an efficient light-harvesting antenna by transferring excitation energy to retinal in the terrestrial xanthorhodopsin from *Salinibacter ruber* (*Sr*XR). Although it also binds to the freshwater xanthorhodopsin Kin4B8, it does not transfer energy to retinal, whereas hydroxylated xanthophylls show high excitation energy transfer (EET) efficiency in this protein. Here, we combine molecular dynamics simulations with polarizable quantum mechanics/molecular mechanics (QM/MM) calculations to construct and characterize the Kin4B8–salinixanthin complex. We obtain a spectroscopically consistent binding model that reproduces the Kin4B8–salinixanthin experimental absorption and circular dichroism spectra and reveals strong electronic coupling between salinixanthin and retinal, comparable to the other xanthophylls. However, energy transfer is strongly suppressed by the red-shift of salinixanthin S_2_ state and its large reorganization energy, which drastically reduce donor–acceptor spectral overlap. These results demonstrate that, in this system, donor vibronic relaxation, rather than geometry or electronic coupling, is the decisive factor suppressing EET.

## Graphical abstract

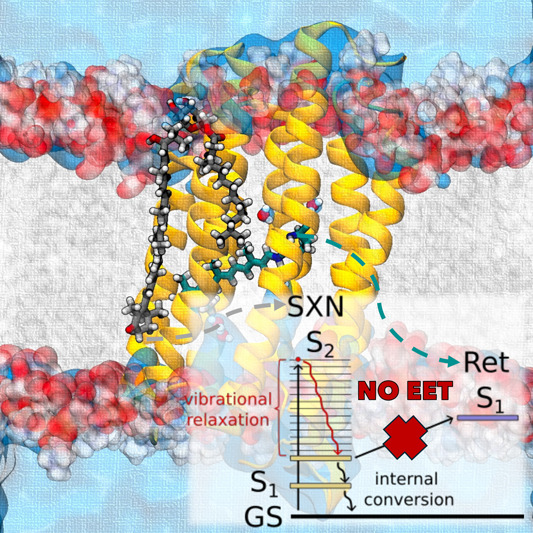

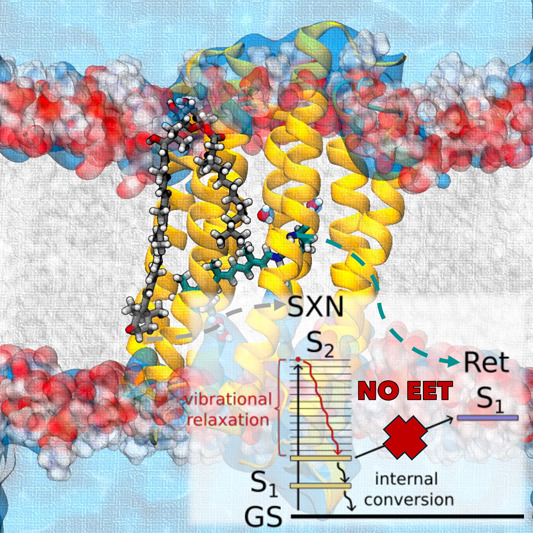

Light-harvesting in nature relies on pigment–protein assemblies that capture solar energy and exploit it for biochemistry with remarkable efficiency.^1^ Among these systems, microbial rhodopsins constitute a widely distributed family of retinal-based photoreceptors that drive ion transport, sensory signaling, and other light-dependent biological functions.^2–6^ While most microbial rhodopsins rely exclusively on retinal for light absorption, a small subset extends its spectral coverage by recruiting carotenoids (Cars) as auxiliary antenna pigments, enabling carotenoid-to-retinal excitation energy transfer (EET).^5,7–16^

Xanthorhodopsin from *Salinibacter ruber* (*Sr*XR) was the first rhodopsin-carotenoid complex to be discovered and remains the canonical example of a carotenoid-assisted photoreceptor.^14,17–27^ Isolated from a terrestrial halophilic bacterium, *Sr*XR employs salinixanthin (SXN) as a natural antenna pigment, achieving carotenoid-to-retinal energy transfer with an efficiency of approximately 40%. The crystal structure of *Sr*XR revealed that the polyene chain of SXN binds noncovalently along the protein, with its 4-keto ring positioned in a pocket adjacent to the retinal chromophore (Figure S1). This arrangement, enabled by a glycine residue (Gly156) near the β-ionone ring of retinal, in contrast to the conserved bulky tryptophan found in many microbial rhodopsins (e.g., bacteriorhodopsin), forms part of the SXN-binding fenestration and places SXN in close proximity to retinal.^19^ Notably, chemical reduction of the 4-keto group (C=O to C–OH) abolishes carotenoid binding and eliminates energy transfer, highlighting its critical role in both structural stabilization and function.^24^ Binding of SXN to *Sr*XR also induces a twist of the 4-keto ring around the C_6_–C_7_ single bond (+82°) and its immobilization, resulting in a slight shift of the absorption spectrum and an increased resolution of vibronic bands.^20,21^ This rigidification of the carotenoid and the resulting sharpening of its vibronic structure enhance spectral overlap and coupling with the retinal chromophore, enabling energy transfer, which in *Sr*XR occurs from the bright S_2_ state of SXN to the S_1_ state of the retinal chromophore on an ultrafast time scale of 66–70 fs.^20,21^ These findings suggested that the presence of a 4-keto group was essential for carotenoid binding in microbial rhodopsins.

This paradigm has been challenged by the discovery of Kin4B8, a proton-pumping xanthorhodopsin from a freshwater *Bdellovibrio* species.^12^ Kin4B8 selectively binds hydroxylated xanthophylls lacking a keto group, such as lutein (LUT) and zeaxanthin (ZEA), that show efficient carotenoid-to-retinal EET.^12,28,29^ The cryo-electron microscopy structure of the Kin4B8-ZEA complex (PDB ID: 8I2Z^12^) reveal a distinctive binding arrangement in which the hydroxyl-bearing ring of the carotenoid inserts into this fenestration, defined by a conserved glycine residue (Gly153 in Kin4B8, corresponding to Gly156 in *Sr*XR), thereby enabling close retinal–carotenoid interactions (Figure 1b). Systematic experimental screening demonstrated EET efficiencies reaching ∼ 56% for these hydroxylated xantophylls (LUT and ZEA), whereas 4-ketocarotenoids, such as canthaxanthin and salinixanthin, were observed to bind but mediate energy transfer poorly in Kin4B8.^12,28^ These findings indicate that pigment chemical identity alone is insufficient to predict antenna efficiency and instead point to a key role of the protein environment.

**Figure 1:**
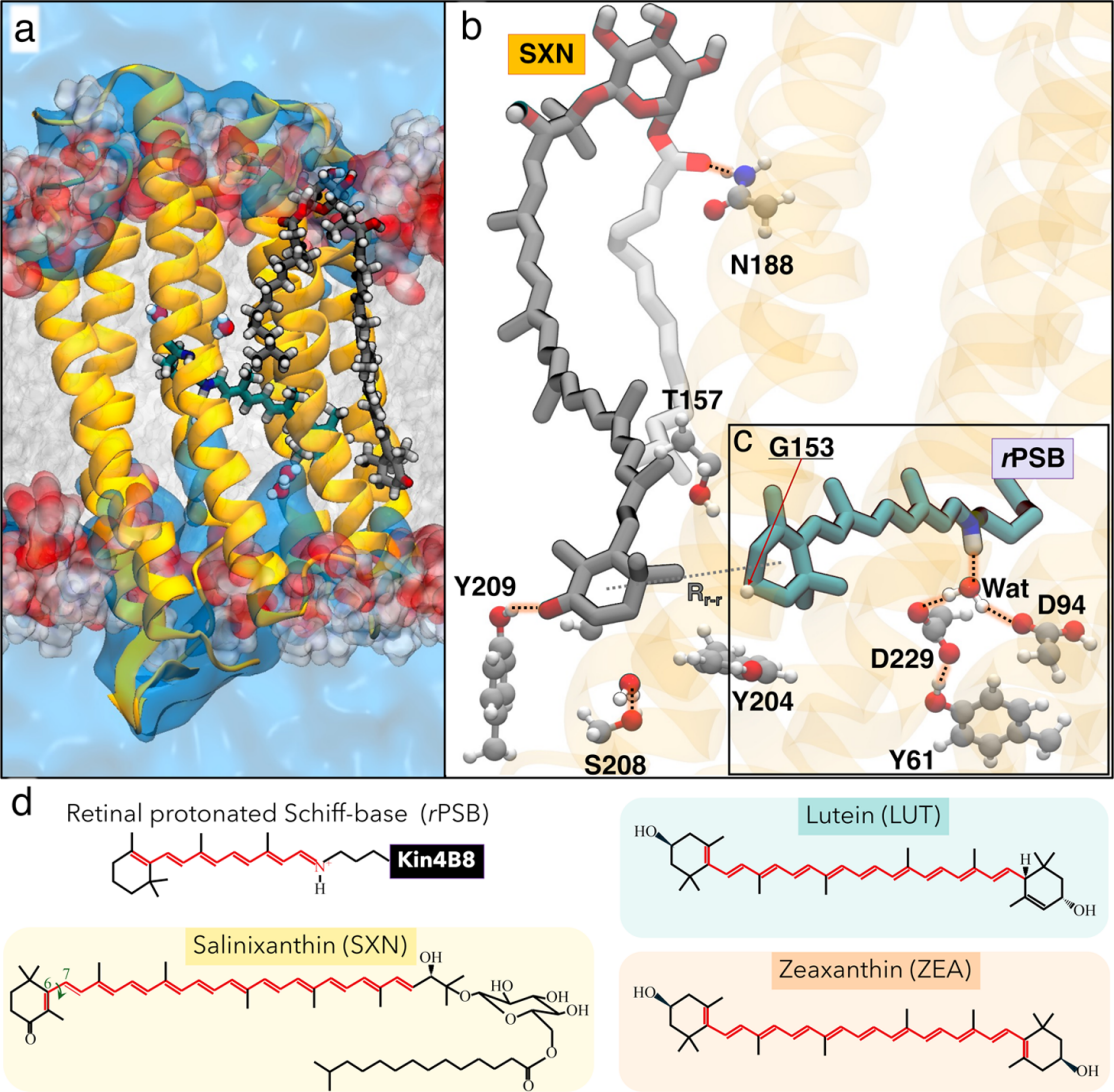
Chromophores binding and fenestration architecture in Kin4B8-salinixanthin. (a) Overall structure of the Kin4B8-salinixanthin model embedded in a lipid bilayer, based on the cryo-EM structure (PDB ID: 8I2Z^12^). The protein is shown in cartoon representation, with the retinal protonated Schiff base (*r*PSB) and salinixanthin (SXN) chromophores depicted in cyan and gray licorice style, respectively. (b) Carotenoid’s fenestration region highlighting key residues: Gly153, Thr157, Tyr204, Ser208, and Tyr209. Gly153 is underlined to indicate its role as the conserved residue defining the carotenoid fenestration in xanthorhodopsins. (c) Counterion environment surrounding the *r*PSB: Asp94 (protonated) and Asp229 (deprotonated), along with Tyr61. (d) Chemical structures of *r*PSB and the xanthophyll carotenoids lutein (LUT), zeaxanthin (ZEA), and SXN. The conjugated polyene chains are highlighted in red. Structural representations are generated using VMD (Visual Molecular Dynamics).^31^

In a recent multiscale computational study, we established a structural and mechanistic framework for understanding efficient EET in Kin4B8 binding hydroxylated xanthophylls.^30^ We demonstrated that ultrafast (<100 fs) and high-efficiency ( > 70%) EET arises from a protein-scaffold chromophore arrangement that maximizes donor–acceptor electronic coupling. In particular, anchoring of the hydroxylated xanthophyll (*i*.*e*., LUT and ZEA) β-ring by a conserved and dynamic hydrogen-bonding network within the fenestration site enforces a binding geometry that optimizes chromophore alignment and tunes both carotenoid and retinal excitation energies.

Despite this emerging mechanistic understanding, a key inconsistency remains unresolved. Salinixanthin, the native and highly efficient antenna pigment of the terrestrial *Sr*XR, binds Kin4B8 as well, as demonstrated by absorption and circular dichroism (CD) spectroscopy, but it does not show any detectable carotenoid-to-retinal energy transfer.^12,28^ Without an experimental molecular structure of the Kin4B8–salinixanthin complex, this behavior cannot be rationalized solely on the basis of pigment binding and remains unexplained. In fact, while the bisignate CD spectrum indicates excitonic coupling between SXN and retinal, this does not reflect in efficient EET.

Which structural factor governs salinixanthin binding to Kin4B8 and suppress excitation energy transfer to retinal in this complex? By constructing and analyzing a three-dimensional model of the Kin4B8–salinixanthin assembly (Figure 1a), we clarify both the binding mode of salinixanthin (Figure 1b) and explain the absence of EET, thereby refining the emerging structure–function paradigm for carotenoid-assisted light-harvesting and defining the limits of protein-mediated control over EET in microbial rhodopsins.

Starting from the cryo-EM structure of Kin4B8 (PDB: 8I2Z),^12^ we constructed an initial model of the Kin4B8–SXN complex using a molecular docking protocol (see Section S1.1). The top-scoring docking pose places SXN within the fenestration site and positions its 4-keto-ring in a configuration closely resembling that observed in the *Sr*XR–SXN X-ray crystallographic structure (PDB ID: 3DDL),^19^ with an overall RMSD of 1.8 Å for the carotenoid heavy atoms (Figure 2a, Figure S1). This pose reproduces key geometric salinixanthin-retinal features previously associated with efficient carotenoid–retinal coupling in *Sr*XR.^19,24,26,32^ Notably, despite the substantial sequence divergence between *Sr*XR and Kin4B8 within the fenestration region (Figure S2), the initial SXN binding mode predicted for Kin4B8 is *Sr*XR-like rather than resembling the orientation adopted by hydroxylated xanthophylls, such as zeaxanthin and lutein, in Kin4B8.

**Figure 2:**
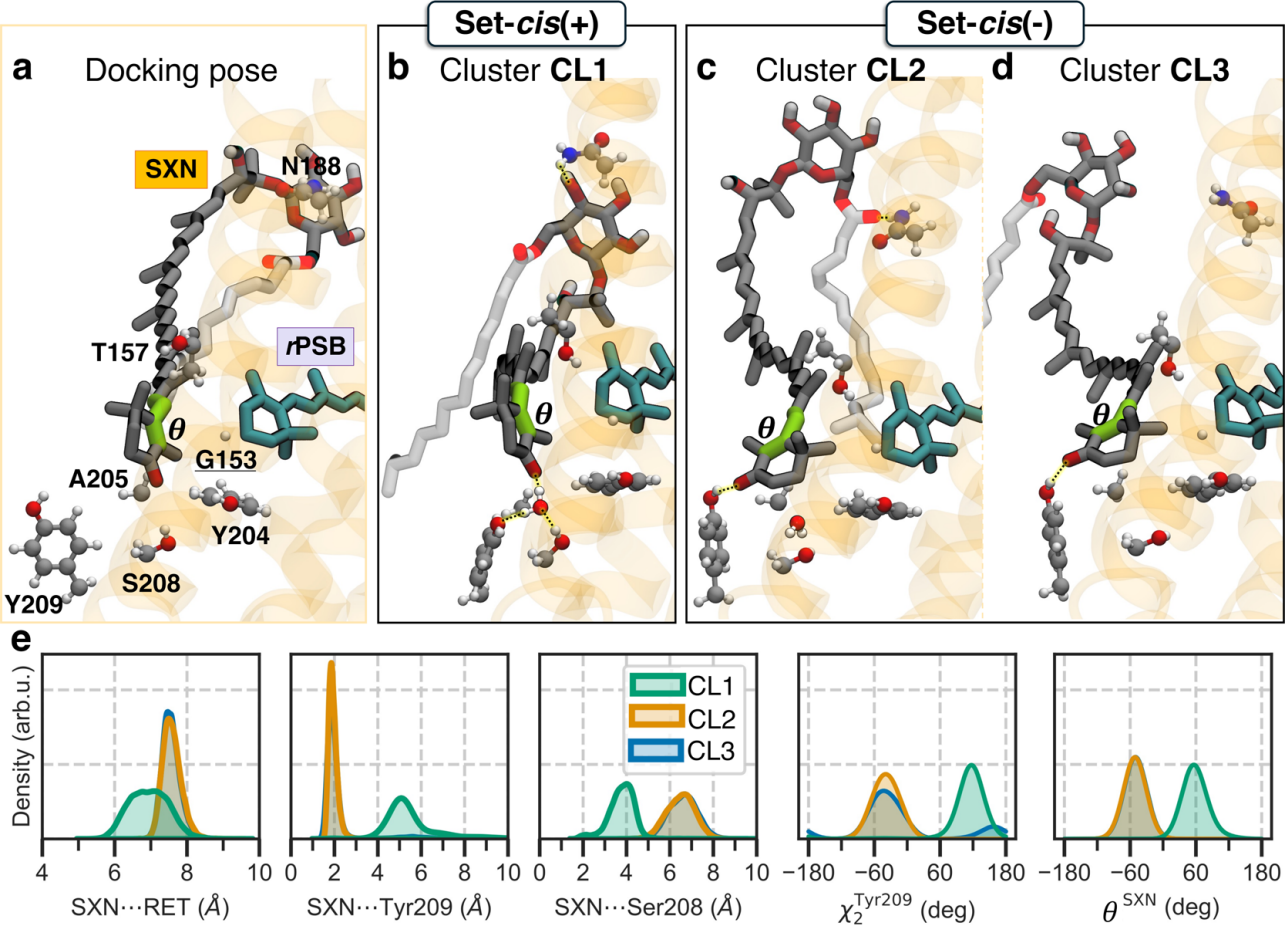
Conformational ensemble of the Kin4B8–salinixanthin (SXN) complex. (a, b, c, d) Representative structures of the initial docking pose and clusters CL1, CL2, and CL3, respectively. Gly153 is underlined to indicate its role as the conserved residue defining the carotenoid fenestration in xanthorhodopsins. (e) Kernel density estimates of selected structural descriptors involving SXN within each cluster (CL1 green, CL2 orange, CL3 blue): distance between the centers of mass of SXN and retinal (RET); minimum distance between SXN and Tyr209; side-chain dihedral angle χ_2_ of Tyr209; and dihedral angle around the C_6_–C_7_ single bond of SXN (θ^SXN^, green tubes). Structural representations are generated using VMD (Visual Molecular Dynamics).^31^

To assess the stability of this *Sr*XR-like binding mode in Kin4B8 and to characterize the conformational landscape accessible to SXN, we perform classical molecular dynamics (MD) simulations in protein membrane (Figure 1a) totaling 4 μs across three independent replicas (two of 1 μs and one of 2 μs; Section S1). The overall structure of the protein and the retinal chromophore remains remarkably stable throughout the simulations. Both the protein backbone and retinal display low RMSDs relative to the initial docked structure (1.3 Å and 1.2 Å, respectively; Figure S4), indicating minimal global or local structural perturbation. Consistent with this stability, analysis of the *r*PSB binding cavity shows that the positions and hydrogen-bonding patterns of the key counterions, Asp94 (protonated) and Asp229 (deprotonated), as well as nearby polar residues, resemble our previous models of the Kin4B8–ZEA and – LUT complexes^30^ (Figure 1c, Figure S7).

Conversely, the salinixanthin binding mode exhibits pronounced heterogeneity. To quantify this, we perform dimensionality reduction using uniform manifold approximation and projection (UMAP),^33,34^ followed by unsupervised clustering with HDBSCAN^35^ based on SXN–protein and SXN–lipid contacts, dihedral angles along the SXN conjugated chain, and the SXN–retinal distance, as described in Section S1.3. This analysis identifies three dominant binding modes, namely CL1, CL2, and CL3, that differ primarily in the orientation of the SXN keto-ring and in the nature of its anchoring interactions within the fenestration site (Figure 2b,c,d; Figure S5). Importantly, all three clusters maintain SXN–retinal ring-to-ring distances in the 6.5–8.0 Å range, comparable to those observed for hydroxylated xanthophylls such as zeaxanthin (7.5 Å) and lutein (7.1 Å) in Kin4B8.^30^ These distances therefore do not explain, by themselves, the absence of EET in Kin4B8-SXN.

In the docking-derived *Sr*XR-like geometry (CL1), the dihedral angle around the C_6_–C_7_ single bond of the salinixanthin $\left(\theta_{C6‐C7}^{SXN}\right)$ adopts a positive s-*cis*(+) of about +60°, positioning the 4-keto group toward the retinal β-ionone ring (Figure 2b). This orientation closely reproduces the relative chromophore arrangement observed in the *Sr*XR–SXN complex and has been proposed to promote efficient energy transfer by optimizing chromophore alignment and transition dipole coupling.^19,24,32^ In Kin4B8, this pose is stabilized by a water-mediated hydrogen bond to Ser208; however, the underlying anchoring interactions differ markedly from those in *Sr*XR, reflecting substantial sequence divergence within the fenestration region. In *Sr*XR, SXN is not stabilized by hydrogen bonding but is instead proposed to be locked in place by a nonpolar interaction between the carotenoid carbonyl and the sulfur atom of Met233.^36^

In contrast, in clusters CL2 (Figure 2c) and CL3 (Figure 2d) SXN adopts a negative s-*cis*(−) twist $\left(\theta_{C6‐C7}^{SXN}\approx -60^{{}^{\circ}}\right)$ , corresponding to a flipped orientation of the SXN keto-ring relative to the docked pose and to the crystallographic *Sr*XR–SXN structure.^19^ In these conformations, the keto-ring faces retinal while the 4-keto group is oriented toward the membrane. CL2 is stabilized by a direct hydrogen bond to Tyr209 and a secondary interaction between the SXN tail carbonyl and Asn188 (Figure 2a), whereas CL3 retains the Tyr209 interaction but replaces the Asn188 contact with transient interactions with the lipid bilayer, consistent with a weaker anchoring. Notably, both CL2 and CL3 closely resemble the binding geometry adopted by hydroxylated xanthophylls such as zeaxanthin and lutein in Kin4B8,^12,28,30^ indicating that the protein scaffold preferentially enforces a conserved anchoring mode largely independent of the carotenoid’s terminal functionalization (Figure 1b). Furthermore, the hydrogen-bonding patterns of the *r*PSB’s counterions, Asp94 (protonated) and Asp229, are preserved across all three SXN cluster binding modes (Figure S7), indicating that the electrostatic environment of the retinal pocket is conserved (Figure 1c).

Although CL1 (the *Sr*XR-like geometry) accounts for the largest fraction of the aggregate conformational ensemble (∼56%), analysis of individual trajectories reveals that this state is metastable. All three MD replicas are initiated from the docked geometry (Figure 2a), yet each underwent spontaneous transitions to CL2 or CL3 during the simulation. As shown in Figure S6, replica 1 converted to CL2 within the first 200 ns, replica 2 persisted in CL1 for approximately 1.8 μs before ultimately converting to CL2, and replica 3 transitioned early and remained in CL3, with the exception of a short-lived transition to CL1 at around 0.3 μs. Overall, no stable back-transitions from either CL2 or CL3 to CL1 are observed, indicating that the s-*cis*(−) conformations represent the thermodynamically preferred binding modes.

Since CL2 and CL3 are nearly indistinguishable in the fenestration region, differing primarily in the orientation of the SXN tail relative to the protein or lipid environment (Figure 2), we treat them as a single representative conformational state, hereinafter referred to as set-*cis*(−), for the spectroscopic and EET analyses. This approximation is justified by the essentially identical local environment experienced by the carotenoid–retinal pair in the two substates, which is expected to have a negligible impact on their electronic properties. Spectra and EET analysis of CL1, from now on called set-*cis*(+), will be presented in the Supporting Information.

These observations demonstrate that, while the Kin4B8 fenestration can initially accommodate an *Sr*XR-like SXN geometry, its intrinsic architecture does not stabilize this configuration. Instead, the protein scaffold actively enforces alternative binding modes that are structurally analogous to those adopted by hydroxylated xanthophylls. This dynamic selection against the *Sr*XR-like geometry provides, in principle, a structural basis for the divergent EET behavior of salinixanthin in Kin4B8 compared to *Sr*XR.

Our simulations refine earlier interpretations of steric constraints in the Kin4B8 binding pocket. A previous work hypothesized that 4-ketocarotenoids such as salinixanthin are unable to adopt an *s-cis* conformation within Kin4B8, thereby precluding efficient energy transfer.^28^ We refine this view by showing that salinixanthin can indeed access *s-cis* (+ or – ) geometries, including an *Sr*XR-like arrangement compatible with EET. However, these states are metastable, whereas alternative binding modes are both structurally preferred and dynamically stable. This conformational bias provides, in principle, a natural explanation for the suppression of efficient energy transfer despite confirmed pigment binding.

Together, these results indicate that variations in SXN binding primarily modulate the carotenoid’s own conformation and its potential for electronic coupling, without significantly altering the structural or electrostatic context of the retinal chromophore. In this sense, the Kin4B8 scaffold provides a robust and invariant retinal environment, while selectively favoring, or dynamically enforcing, carotenoid conformations that are incompatible with efficient energy transfer.

Structural feasibility alone cannot fully account for the absence of EET in the Kin4B8–SXN complex. We therefore apply our previously established computational framework^30^ for carotenoid–retinal energy transfer on both set-*cis*(−) and set-*cis*(+). This framework enables validation of the proposed binding modes against experimental absorption and CD spectra, followed by a kinetic assessment of EET suppression in the same conformations. Details on exciton and vibronic parameters are provided in Section S1.

The excitation energies, calculated at ground-state optimized QM/MM geometries (see Methods Section), corresponding to the S_0_ → S_1_ transition of *r*PSB and the S_0_ → S_2_ transition of salinixantin, are reported in Figure 3a, together with reference values for Kin4B8–ZEA and – LUT.^30^ A pronounced red shift of the mean Car S_2_ excitation energy is observed for SXN, following the trend LUT (2.76 eV) > ZEA (2.68 eV) > SXN (2.53 eV). This red-shift is primarily intrinsic, arising from the extended conjugation through the keto group, as confirmed by vacuum calculations at the same geometries (Figure 3b) that leads to a systematic blue-shift of the carotenoid S_2_ energies for all pigments, while preserving the same energetic ordering (LUT > ZEA > SXN). This confirms that the relative red-shift of SXN is dominated by intrinsic electronic structure effects, with the protein environment providing an additional, largely uniform (Figure S9), stabilization.

**Figure 3:**
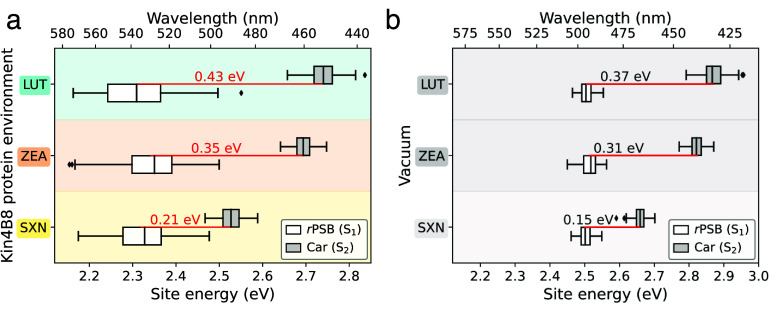
Carotenoid–retinal site energies in Kin4B8–Car complexes and in a vacuum. Box plot of the Car S_2_ and *r*PSB S_1_ excitation energies computed on the ground-state-optimized geometries in the (a) Kin4B8–Car protein environments and (b) in the gas phase at the same optimized geometries. Boxes indicate the interquartile range (25th–75th percentiles), with the median shown as a horizontal black line; whiskers extend to 1.5 times the interquartile range. Horizontal red lines denote the Car–*r*PSB energy gaps (in eV), following the order LUT-*r*PSB > ZEA-*r*PSB > SXN-*r*PSB.

The retinal S_1_ excitation energy remains nearly invariant across all systems, exhibiting only minor fluctuations irrespective of the identity of the bound carotenoid. As a consequence, the energy gap between the carotenoid S_2_ donor state and the retinal S_1_ acceptor state is reduced in the Kin4B8–SXN complex (Figure 3a). As the EET rate depends on the donor–acceptor energetic separation, we anticipate that this narrowing will affect the EET efficiency.

We then evaluated the electronic coupling between the SXN­(S_2_) and *r*PSB­(S_1_) bright transitions, computed from the Coulomb interaction between their transition densities, obtained from TD-DFT calculations within a polarizable embedding QM/MM framework^37–40^ (Section S1.4). Table 1 reports the average coupling obtained from the dominant set-*cis*(−) binding modes, along with the reported data for Kin4B8–ZEA and – LUT.^30^ The SXN–retinal coupling is slightly reduced relative to ZEA and LUT, yet remains within the same order of magnitude, indicating that electronic coupling alone is unlikely to be the limiting factor for energy transfer in the SXN complex. Notably, the coupling computed for the set-*cis*(+) *Sr*XR-like binding mode (291 cm^–1^) is comparable to the ZEA and LUT values, further excluding weak electronic coupling as the primary origin of the suppressed EET. This conclusion is further supported by structural considerations. In our previous work, we identified the carotenoid–retinal ring–to-ring distance (*R*_r–r_) as a reliable structural descriptor of coupling strength in the rhodopsin system, particularly in regimes where the dipole–dipole approximation breaks down due to short intermolecular separations or distorted geometries.^30^ As shown in Table 1, *R*_r–r_ is nearly identical across all complexes, indicating that Kin4B8 accommodates the carotenoid ring within the same fenestration in all cases. This conserved geometry is therefore fully compatible with effective electronic coupling, reinforcing the conclusion that coupling strength is not the limiting factor for EET in the Kin4B8-SXN complex.

**Table 1:** Electronic, Excitonic, and Structural Parameters Used in the EET Kinetic Model for the Kin4B8 Complexes^a^

	Exc. energy (nm)		Coup. (cm^–1^)	|μ| (D)^b^				
Car	*r*PSB^a^	Car	*r*PSB-Car	*r*PSB	Car	R_c‑c_ (Å)	R_r‑r_ (Å)	$\lambda_{\mathrm{reorg}}^{\mathrm{Car}}$ (cm^–1^)
SXN set-*cis*(−)^c^	535	491	248	9.5	13.6	16.7	7.5	3593
SXN set-*cis*(+)^c^	545	481	291	9.5	13.5	16.7	7.0	3593
ZEA [ref 30]	530	460	270	9.5	12.4	12.5	7.5	1458
LUT [ref 30]	537	453	283	9.5	11.9	12.8	7.1	1787

a Structural parameters were derived from QM/MM-optimized geometries.

b Average values. Red-shifted by 0.2 eV, according to ref. 30.

c Transition dipole moment in Debye.

d Average $\theta_{C6‐C7}^{SXN}$ for QM/MM optimized structures: −47° for set-*cis*(−) and +62° for set-*cis*(+).

Another key factor modulating the EET is the vibrational reorganization energy of the involved chromophores, namely λ_reorg_. It quantifies the energetic relaxation of the donor’s excited state and determines the energy of its lower vibronic states, thereby modulating spectral overlap with the retinal acceptor. The reorganization energy for each Car computed in the Kin4B protein environment are reported in Table 1. Interestingly, the value for salinixanthin ( $\lambda_{\mathrm{reorg}}^{\mathrm{SXN}}$ = 3593 cm^–1^) is more than twice that of both zeaxanthin ( $\lambda_{\mathrm{reorg}}^{\mathrm{ZEA}}$ = 1458 cm^–1^) and lutein ( $\lambda_{\mathrm{reorg}}^{\mathrm{LUT}}$ = 1787 cm^–1^), suggesting the pronounced reorganization on the bright S_2_ state of the former, which in turn might have an effect on the EET properties.

The simulated absorption spectrum of Kin4B8–SXN (Figure 4a, see Section S2) reproduces the broad, unstructured band centered at 500 nm observed experimentally (Figure 4b, Figure S10), in contrast to the vibronically resolved spectra of ZEA and LUT.^12,30^ This broadening arises from the more intense low-frequency spectral density of SXN (see Figure S8a). Comparison with the vacuum SXN spectral density (Figure S8c) indicates that the increased absorption broadening arises predominantly from environmental effects rather than intrinsic properties of SXN.

**Figure 4:**
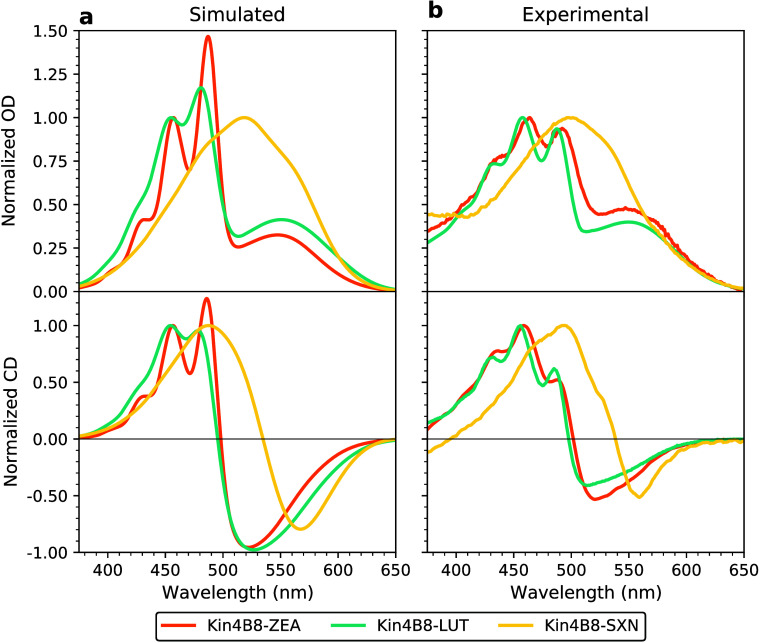
(a) Simulated and (b) experimental^12^ spectra of different Kin4B8–Car complexes. Upper panels show absorption spectra, while CD is shown in the lower panels. Different carotenoids are shown: salinixanthin (SXN, yellow lines), while previously reported spectra for Kin4B8–lutein (LUT, turquoise) and Kin4B8–ZEA (ZEA, orange) are included for comparison. For the Kin4B8–SXN complex, we show results from the set-*cis*(−), while spectra for set-*cis*(+) are presented in Figure S10 and Figure S11.

As a more stringent test of the binding mode, we simulate the CD spectrum, whose bisignate shape was used as a key diagnostic feature for identifying salinixanthin binding within the Kin4B8 rhodopsin complex.^12,28^ Our CD simulated on the set-*cis*(−) ensemble recovers both the biphasic shape and the spectral positions of the experimental positive and negative bands (Figure 4a,b). Decomposition of the contributions of individual pigments to spectra reveals that the positive CD band originates from salinixanthin, while the negative signal arises primarily from the retinal chromophore (Figure S11). As in the previously characterized Kin4B8–ZEA and – LUT complexes,^30^ the overall CD profile is dominated by excitonic interactions between the pigments, justifying a strong SXN-*r*PSB electronic coupling similar to ZEA and LUT. Remarkably, the relative intensity between the positive and negative peaks is better reproduced in the set-*cis*(−), compared to set-*cis*(+) (see Figure S11).

We are now in a position to explicitly examine excitation energy transfer in the Kin4B8 complexes. Energy transfer rates from the carotenoid donor (S_2_) to the retinal acceptor (*r*PSB, S_1_) follow the Fermi golden rule,

1 $$k_{DA}=2\frac{|V_{DA}{|}^{2}}{\hbar^{2}}J_{DA}$$

where *V*_*DA*_ is the donor–acceptor electronic coupling and *J*_*DA*_ is the spectral overlap between donor emission and acceptor absorption. We thus computed the spectral overlap as detailed in Section S2. This approach was previously validated for Kin4B8–ZEA and – LUT.^30^

While lutein and zeaxanthin exhibit substantial spectral overlap with the retinal absorption (∼1.5 eV^–1^; Figure 5a), consistent with the high spectral overlap estimated from experimental spectra (∼1.2 eV^–1^),^29^ in Kin4B8–SXN the spectral overlap is strongly reduced (0.06 eV^–1^, Figure 5b). This reduction arises from two concurrent effects: (i) the reduced energy gap between the SXN S_2_ donor state and the retinal S_1_ acceptor state (see Section S5 for a quantitative analysis), and (ii) the substantially larger reorganization energy of salinixanthin ( $\lambda_{\mathrm{reorg}}^{\mathrm{SXN}}∼$ 3500 cm^–1^), compared to lutein and zeaxanthin ( $\lambda_{\mathrm{reorg}}^{\mathrm{ZEA}/\mathrm{LUT}}∼$ 1500 cm^–1^). To disentangle these contributions, we constructed two artificial models (Section S5, Figure S12). Restoring the LUT–*r*PSB energy gap while retaining the SXN spectral density increases the overlap only modestly (0.48 eV^–1^; 36% efficiency). In contrast, replacing the SXN spectral density with that of LUT, while keeping the SXN site energies unchanged, recovers a large overlap (1.7 eV^–1^) and near-LUT efficiency (66%). This demonstrates that the large reorganization energy, not the energy gap, is the dominant factor suppressing EET. To understand the origin of such a large reorganization energy in the Kin4B8 environment, we recalculated the spectral density for SXN in vacuum (See Section S1.5). This calculation resulted in a reorganization energy of 2747 cm^–1^, which is larger than that of LUT/ZEA-Kin4B8 environment (∼1500 cm^–1^), but smaller than that of SXN-Kin4B8 (∼3500 cm^–1^), indicating that the large reorganization energy computed here arises from both intrinsic and protein-induced effects.

**Figure 5:**
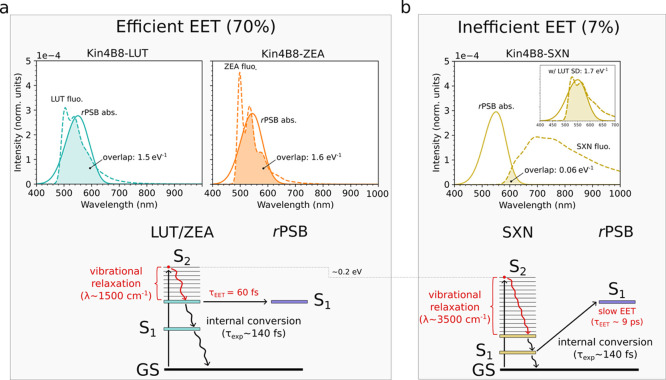
Energy transfer and spectral overlap in Kin4B8 complexes binding different carotenoids: lutein (LUT), zeaxanthin (ZEA), and salinaxanthin (SXN). (a) Efficient EET for ZEA and LUT. (b) Inefficient EET for SXN. Upper panels: computed spectral overlap between carotenoid calculated fluorescence (fluo.) and calculated *r*PSB absorption (abs.). Using the LUT spectral density (SD) for SXN recovers a large spectral overlap and efficient EET (inset). Simulated absorption and fluorescence spectra were computed for the chromophores in the Kin4B8 protein environment. Details on the calculation of fluorescence and absorption spectra are given in Sec. S1.5. Lower panels: vibronic Jablonski diagrams showing the ground state (GS), the carotenoid S_2_ state, and the *r*PSB S_1_ state. Vibrational relaxation on the carotenoid S_2_ manifold is indicated together with the associated reorganization energy. Calculated EET times for the Car S_2_ → *r*PSB S_1_ state are indicated in red. Measured S_2_ internal conversion times to the ground state through the S_1_ state are indicated in black. The relaxation time measured from reduced-retinal LUT-binding Kin4B8 (τ = 140 fs)^12^ was adopted for all carotenoids. The EET efficiencies are calculated according to eq. S19.

We then calculated the EET rates using the obtained couplings and spectral overlaps in eq. 1. The large spectral overlaps of lutein and zeaxanthin result in fast transfer rates, corresponding to EET times of ca. 60 fs,^30^ whereas the much smaller overlap of SXN results in a markedly slower EET time of about 9 ps (see Figure 5a,b, lower panels). As the Car–*r*PSB couplings are comparable across the Kin4B8–LUT, – ZEA, and – SXN complexes (248–283 cm^–1^; Table 1), differences in EET kinetics virtually depend only on the spectral overlap.

The Car­(S_2_) → *r*PSB­(S_1_) EET pathway competes with the intrinsic ultrafast relaxation of xanthophylls, dominated by S_2_ → S_1_ internal conversion on a ∼ 100 fs time scale,^20,21,23,27,41^ followed by relaxation to the ground state (S_1_ → GS) (Figure 5a,b, lower panels). To estimate the EET efficiency (See Section S1 and eq. S19), we assume a lifetime τ_*D*_ = 140 fs for the isolated donor, corresponding to the measured S_2_ lifetime of lutein in Kin4B8 when retinal-mediated EET is chemically suppressed.^12^ Without specific measurements for Kin4B8-SXN, this choice provides a consistent reference time scale to quantify the competition between EET and ultrafast internal conversion. This results in an efficiency of ∼ 7% for Kin4B8–SXN, much lower than the ∼ 70% efficiency calculated for Kin4B8–LUT/ZEA. Considering that salinixanthin exhibits an even faster S_2_ relaxation in *Sr*XR,^20,21,23,27,41^ the results reported here for Kin4B8–SXN should be regarded as upper limits for the EET rates and efficiencies. Using a kinetic model (see Section S2), we simulated the excited-state population dynamics for the Kin4B8–SXN complex (Figure S13c), and compared them to LUT and ZEA (Figure S13a,b). Upon selective excitation of the SXN S_2_ state at *t* = 0, the population predominantly relaxes to the ground state and reaches a steady distribution within ∼ 0.6 ps, while the retinal S_1_ population remains below 0.1.

Importantly, our results are fully consistent with the experiments. First, the EET efficiency determined for Kin4B–LUT (∼70%) is consistent with the high EET efficiency measured experimentally for Kin4B8–LUT (∼55%),^12,28,30^ although slightly overestimated. Second, fluorescence excitation measurements on Kin4B8–SXN have not detected any retinal fluorescence upon SXN excitation,^12,28^ in agreement with our poor SXN-to-*r*PSB EET efficiency in Kin4B8–SXN. Interestingly, despite the strongly suppressed EET, the Kin4B8–SXN complex still exhibits a pronounced excitonic CD signal (see Figure 4). In fact, while the electronic coupling between SXN and retinal remains strong, the large Stokes shift associated with SXN displaces the donor emission away from the retinal absorption, thereby severely reducing the spectral overlap required for efficient energy transfer.

The Kin4B8 fenestration accommodates both hydroxylated and 4-keto xanthophylls with comparable geometries and electronic couplings, yet efficient EET is observed only for the hydroxylated species. The suppression of EET in Kin4B8–SXN does not arise from unfavorable binding geometry or weak coupling, but from the excited-state properties of SXN tuned by the protein environment: a red-shifted S_2_ state and, predominantly, a large reorganization energy that suppresses spectral overlap with retinal. Even the metastable *Sr*XR-like set-*cis*(+) binding mode yields only modest EET (spectral overlap 0.15 eV^–1^; ∼ 20% efficiency), insufficient to compensate. This behavior indicates that the differences in EET efficiency between systems such as Kin4B8 and *Sr*XR originate from how the protein environment balances structural rigidity and low-frequency fluctuations, thereby modulating reorganization energy and spectral overlap.

Our results demonstrate that the fenestration architecture of microbial rhodopsins plays a decisive role in determining both carotenoid binding selectivity and excitation energy transfer efficiency. While the xanthorhodopsin *Sr*XR fenestration is structurally optimized to accommodate salinixanthin in a geometry that supports efficient EET, the Kin4B8 fenestration selectively favors hydroxylated xanthophylls. In Kin4B8, salinixanthin can bind in multiple conformations, but only one is consistent with the experimental absorption and CD spectra. Despite this spectroscopically validated binding mode and the presence of strong excitonic coupling, kinetic simulations confirm the absence of efficient carotenoid-to-retinal energy transfer, in agreement with experimental observations.^12,28^ These differences may reflect adaptation to distinct ecological niches, with Kin4B8, originating from a freshwater environment, favoring flexible light-harvesting through selective binding^12,28^ and pH-dependent tuning of EET efficiency,^29^ in contrast to *Sr*XR from *Salinibacter ruber*, which operates in hypersaline conditions.

Importantly, the lack of EET observed in Kin4B8-SXN does not stem from unfavorable binding geometry or weak electronic coupling, but from intrinsic excited-state properties of salinixanthin in the Kin4B8 environment, including a red-shifted S_2_ state and increased nuclear reorganization that suppress spectral overlap with retinal. These results highlight that effective antenna design requires simultaneous control of chromophore chemistry, protein-imposed geometry, and excited-state energetics. By identifying structural and dynamical features that actively suppress energy transfer, this work provides actionable design principles for engineering rhodopsin–carotenoid assemblies with tunable light-harvesting functionality, extending beyond natural systems toward bioinspired photonic materials and optogenetic architectures.

## Methods and Computational Details

Starting from the cryo-EM structure of Kin4B8 [8I2Z],^12^ the Kin4B8–SXN complex was constructed by molecular docking and subsequently refined through multimicrosecond classical MD simulations in an explicit lipid bilayer (Section S1), using the Amber 22 package.^42^ Representative snapshots were extracted from MD trajectories for subsequent QM/MM calculations, yielding 79 structures for the set-*cis*(−) ensemble and 30 structures for set-*cis*(+). These geometries are optimized using a QM/MM scheme with electrostatic embedding in which the QM region comprised the SXN–*r*PSB dimer, treated at the density functional theory (DFT) level with the CAM-B3LYP^43^ functional and the 6–31G­(d)^44^ basis set. Vertical excitation energies were then computed using time-dependent DFT (TD-DFT)^45^ at the CAM-B3LYP/6–31+G­(d) level within a polarizable embedding QM/MMPol scheme.^37,46–52^ This methodology is identical to that previously introduced by us in ref 30 and has been shown to provide a reliable description of carotenoid and retinal excited-state energetics in Kin4B8 (more details are given in Section S1.4). Exciton couplings were calculated by direct integration of transition densities obtained from QM/MMPol calculations.^37,39^ All QM/MM calculations in this work were run using a locally modified version of Gaussian.^53^

Spectral densities were calculated using the vertical gradient approach.^39,54,55^ Absorption and circular dichroism spectra were calculated using the Full Cumulant Expansion theory.^56^ EET rates were calculated using the Förster EET theory.^39,57,58^ The spectra and EET rates, as well as the population dynamics, were computed with the Python-based pyQME software package^59,60^ developed in our group.

## Supplementary Material



## Notes

Computational details (system preparation, protonation states, docking, MD, QM/MM­(Pol) DFT, spectral densities); theory of exciton Hamiltonian, excitation energies, electronic and vibronic couplings, spectroscopy, and EET kinetics; EET population dynamics; models for the effects of salinixanthin excitation energy and vibronic reorganization on EET; and supplementary figures (PDF)
