# Supplementary material for: Vibronic Reorganization Suppresses Salinixanthin-to-Retinal Energy Transfer in the Freshwater Kin4B8 Xanthorhodopsin
Source: J Phys Chem Lett. 2026 Apr 7;17(15):4551–60. doi: 10.1021/acs.jpclett.6c00507 (PMC13093654; doi:10.1021/acs.jpclett.6c00507)
Supplement: Supplementary file 1 [file jz6c00507_si_001.pdf]

Supporting Information: Vibronic Reorganization Suppresses Salinixanthin-to-Retinal Energy Transfer in the freshwater Kin4B8 Xanthorhodopsin

Giacomo Salvadori,[†,¶] Piermarco Saraceno,[‡,¶] Chris John,[‡] Lorenzo Cupellini,[‡] and Laura Pedraza-González[*,‡]

†*Institute for Computational Biomedicine (INM-9), Forschungszentrum Jülich, 52428 Jülich,Germany*

‡*Dipartimento di Chimica e Chimica Industriale, Università di Pisa, via G. Moruzzi 13, 56124 Pisa, Italy*

¶*These authors contributed equally to this work.*

E-mail: laura.pedraza@unipi.it

Contents

S1 Methods **S3**

S1.1 System preparation and molecular docking S3

S1.2 Molecular dynamics simulations S5

S1.3 Conformational analysis S7

S1.4 QM/MM(Pol) DFT calculations S10

S1.5 Spectral density calculations S12

S2 Theory **S14**

S2.1 Exciton Hamiltonian S14

S2.2 Coupling to vibrations S14

S2.3 Spectroscopy S15

S2.4 EET rates and kinetic model S16

S3 Color tuning analysis **S19**

S4 Decomposition of absorption and circular dichroism spectra **S20**

S5 Effect of salinixanthin excitation energy and its vibronic reorganization energy on EET **S22**

S6 Population dynamics **S24**

References **S25**

S1 Methods

S1.1 System preparation and molecular docking

The Kin4B8 rhodopsin is prepared starting from the cryo-EM structure (PDB 8I2Z[1]). Protonation states are assigned at pH 5.2 using the ARM protocol,[2] yielding Asp(H)94, Asp229, Glu(H)105, and His60 in the ϵ-tautomer form (HIE, protonated at $N\epsilon$ and deprotonated at $N\delta$). The structure is subsequently energy-minimized using the AMBER ff14SB force field.[3] Since no experimental Kin4B8–SXN structure is available, the SXN binding mode is obtained through molecular docking with AutoDock 4.2.2.[4] A grid of $120 \times 120 \times 120$ points (0.375 Å spacing) covering the entire protein and fenestration region is used. Two hundred Lamarckian GA runs are performed and a maximum of 25 000 000 energy evaluations; all other docking parameters are kept at their default values. The best poses are selected based on docking scores and structural consistency with the Xanthorhodopsin–SXN X-ray structure (PDB 3DDL[5]). The comparison is shown in Figure S1.

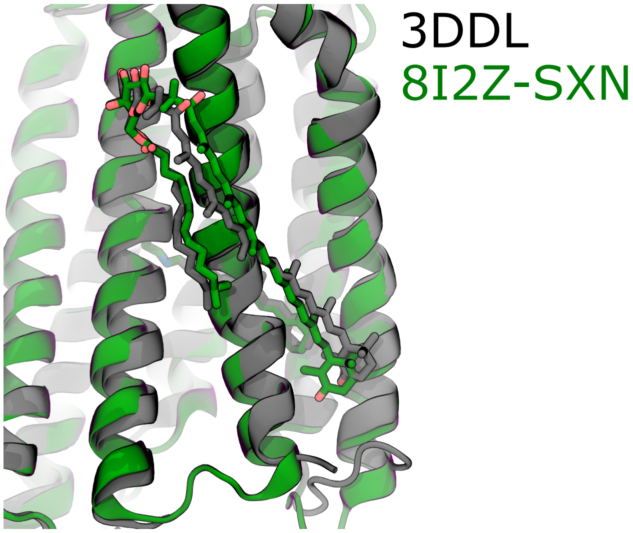

Figure S1: Superimposition of the XR–SXN structure (PDB 3DDL, gray) and the docked SXN pose in Kin4B8 (green).

```
3DDL      MLQELPTLTPGQYSLVFNMFSFTVATMTASFVFFVLARNNVAPKYRISMMVSALVVFIAG  60
8I2Z      --MSATTLTLQQFSTVYNMLSFAVASMLGAFAFFVMGRKIVGPKYRLALVVSSLVVLIAG  58
             .  ***  *:* *:**:**:**:* .:*.***:.*: *.****::::**:***:***

3DDL      YHYFRITSSWEAAYALQNGMYQPTGELFNDAYRYVDWLLTVPLLTVELVLVMGLPKNERG  120
8I2Z      YHYWRIMGSWTAAYALKDGMYVPTGEPFNDAYRYVDWLLTVPLLLTELVLVMKLKKE-SG  117
          ***:** .** *****::*** **** ***************** .****** * *:  *

3DDL      PLAAKLGFLAALMIVLGYPGEVSENAALFGTRGLWGFLSTIPFVWILYILFTQLGDTIQR  180
8I2Z      SVLAKLILAAIAMIALGYPGEISNPESQAGARLMWGVLSTVPFLYILYVLWVRLGDAIGE  177
           : *** : *  **.******:*:  :  *:* :**.***:**::***:*:.:***:* .

3DDL      QSSRVSTLLGNARLLLLATWGFYPIAYMIPMAFPEAFPSNTPGTIVALQVGYTIADVLAK  240
8I2Z      HPAKVQVLLKNTRYLILLTWGFYPIVYAMGSY----GWLGGAGSVVAVQVGYSIADVTAK  233
          : ::*..** *:* *:* *******.* :          .  *::**:****:**** **

3DDL      AGYGVLIYNIAKAKSEEEGFNVSEMVEPATASA     273
8I2Z      ALYGVMIFAIAYAKSEADGSLPA----------     256
          * ***:*: ** **** :*   :
```

Figure S2: **Sequence alignment** of *Sr*XR (3DDL) and Kin4B8 (8I2Z) rhodopsin proteins. Amino acids are represented by their single-letter codes. Asterisks (*) denote identical residues, while colons (:) indicate conservation between groups of strongly similar properties.

S1.2 Molecular dynamics simulations

Kin4B8-SXN complex is embedded in explicit POPC lipid bilayers using the CHARMM-GUI website[6] with dimensions of $75 \times 75\text{Å}^2$ in the membrane plane. The system is solvated with TIP3P water molecules[7] extending at least 20Å from the membrane surface and neutralized with 0.15 M KCl to approximate physiological ionic strength. We used the AMBER ff14SB forcefield[3] to describe the protein, and the lipid21 forcefield[8] for the lipids. The membrane has been first equilibrated following the steps summarized in Table S1.

Table S1: Membrane equilibration procedure. "SD" and "CG" stand for "steepest descent" and "conjugate gradient", respectively. "Bilayer" means that the constraint is applied to the non-hydrogen atoms of the lipid bilayer.

	Time	Thermodynamic ensemble	Harmonic potential	Temperature
Minimization	4000 SD<br>6000 CG	-	10 kcal $mol^{-1}\text{Å}^{-2}$<br>bilayer	-
	4000 SD<br>6000 CG	-	-	-
Heating	10 ps	NVT	10 kcal $mol^{-1}\text{Å}^{-2}$<br>bilayer	0K→100K
	100 ps	NPT	10 kcal $mol^{-1}\text{Å}^{-2}$<br>bilayer	100K→310K
Equilibration	5.0 ns	NPT	-	310K
Production	120.0 ns	NPT	-	310K

Afterwards, to fit the Kin4B8-SXN complex into the membrane, we used an in-house tool that remove any lipid or water molecule within 0.5–2 Å of the receptor.

Forcefield parameters for *r*PSB are adapted from previous work.[9] To develop force field parameters for salinixanthin, we employ a fragment-based approach dividing the molecule into three distinct regions: the polyene chain, the glycosyl moiety, and the acyl ester tail

(Figure S3). For the polyene chain (Figure S3C), we adapted validated parameters from literature.[10] The acyl tail and the glycosyl moiety (Figure S3A,B) are parametrized using the generalized AMBER force field (GAFF).[11] Ligand partial charges for every fragment are computed using the RESP protocol[12] at the B3LYP[13,14]/6-311G(d,p) level of theory on top of B3LYP/6-31G(d,p)-optimized geometries in implicit water solvent (IEF-PCM) to account for long-range solvation effects.[15–17] The glycosyl fragment required special consideration due to its conformational flexibility in aqueous solution. We conduct QM(DFTB3)/MM molecular dynamics simulations[18,19] of the methyl-capped glycosyl fragment in TIP3P water to sample its conformational space, identifying 22 representative structures through clustering analysis. RESP charges are then derived from B3LYP/6-311G(d,p) calculations on these conformations with multiconformational resp performed with PyRESP.[20] All quantum-chemical calculations are performed using Gaussian16.[21]

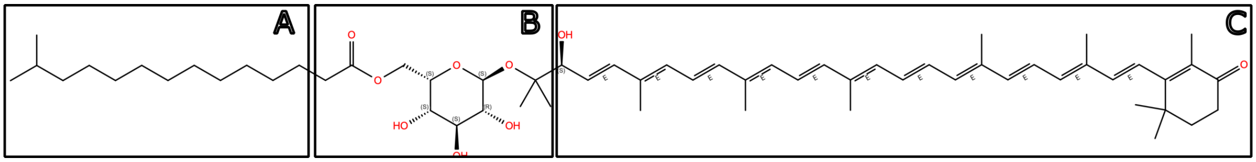

Figure S3: **Fragments used for SXN parametrization**: acyl tail (A), glycosyl moiety (B), and polyene chain (C).

A first minimization is performed only on the residues within 2.0 Å of the protein. Then, we minimized all the water molecules and the hydrogen atoms, and finally the entire system without constraints. A 5 ps simulation in the NVT ensemble followed by a 100 ps simulation in the NPT ensemble are used to heat the system first to 100 K, and then to 310 K, with the protein constrained by a 10 kcal mol^{-1}Å^{-2} harmonic restraint in both cases and a timestep of 1fs. The box equilibration step is performed in the NPT ensemble by gradually releasing the restraints to 0.4 kcal mol^{-1}Å^{-2} in 5 ns with a timestep of 2fs. An additional 50 ns simulation is performed to equilibrate the loops and other mobile regions of the protein, restraining only the backbone of the transmembrane helices by a 0.4 kcal mol^{-1}Å^{-2} harmonic restraint. The production simulations are performed without any constraint for 1 μs in the NPT ensemble

with a timestep of 2 fs. We run NPT simulations run with the Berendsen barostat to maintain a pressure of 1 bar with a semiisotropic pressure scaling and a pressure relaxation time of 2.0 ps. The temperature is held constant at 310 K using the Langevin thermostat. The SHAKE algorithm is used in all simulations along with a 2 fs time step. Particle-mesh Ewald electrostatics with a 12 Å non-bonded cutoff is used. Simulations are carried out with the `PMEMD` implementation of `SANDER`, release 22.[22–25]

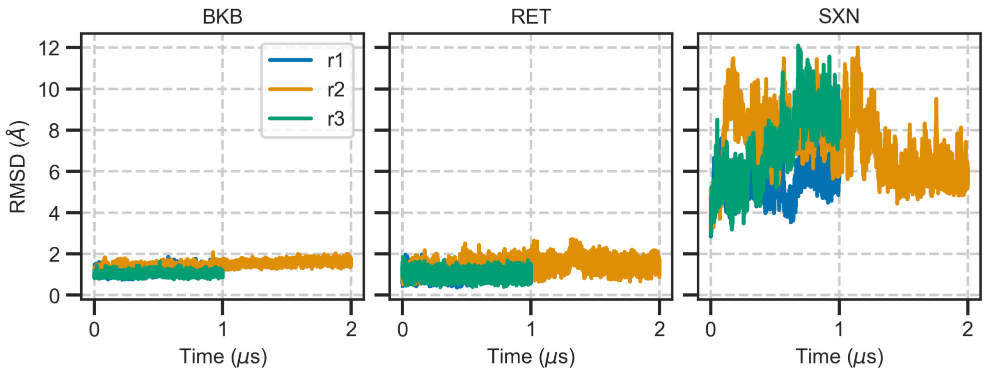

Figure S4: **Root Mean Square Deviation (RMSD) of Protein Kin4B8-SXN and associated chromophores**. Time-dependent RMSD values for three independent classical molecular dynamics replicas (r1, r2, and r3). Analysis includes the protein backbone, retinal (RET), and salinixanthin (SXN).

S1.3 Conformational analysis

To characterize the conformational ensemble of the Kin4B8-SXN complex, we extract geometric descriptors for each MD snapshot (1 frame every 50 ps, for a total of 80000 frames), including distances between SXN and selected residues (Tyr207, Ser206, Asn186, retinal), the SXN–retinal center-of-mass distance, the minimum distance between the SXN 4-keto oxygen and water molecules, and dihedral angles d1, d2, and two Tyr207 side-chain torsions (Figure S5). The dihedrals are considered using the sine and cosine components. All the features are then standardised. The final dataset comprises 80000 structures per 17 features. To reduce the dimensionality of the problem, we use UMAP[26] with n_components=2, n_neighbors=200, min_dist=0.2, random_state=2026, and metric=euclidean. On top of

that, we have applied a hierarchical HDBSCAN algorithm with min_samples=100 and min_cluster_size=5000 finally obtaining three clusters (Figure S5), which are subsequently used for QM/MM calculations and exciton–vibronic modeling.

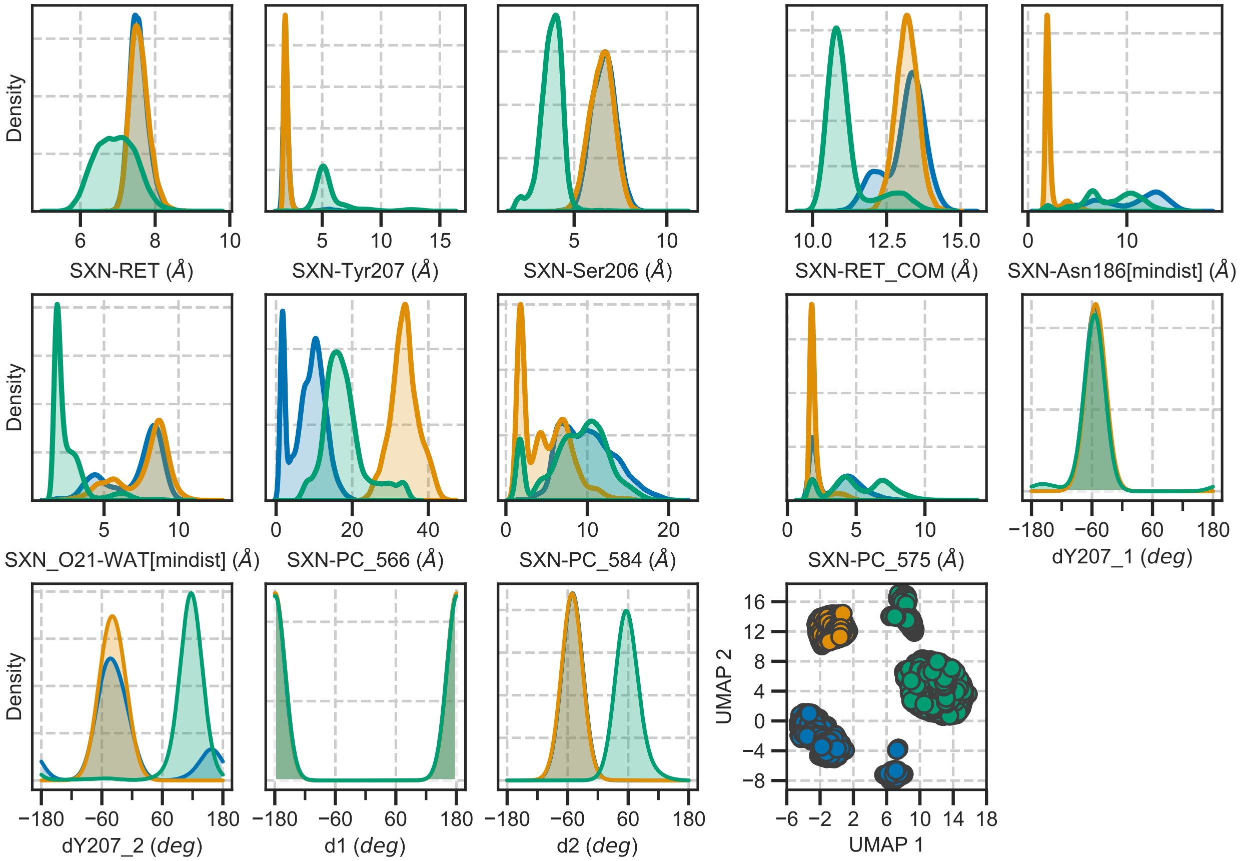

Figure S5: **Feature** distributions and **UMAP projection** of the Kin4B8–SXN conformational ensemble. Colors indicate the three HDBSCAN clusters: CL1 (green), CL2 (orange), and CL3 (blue).

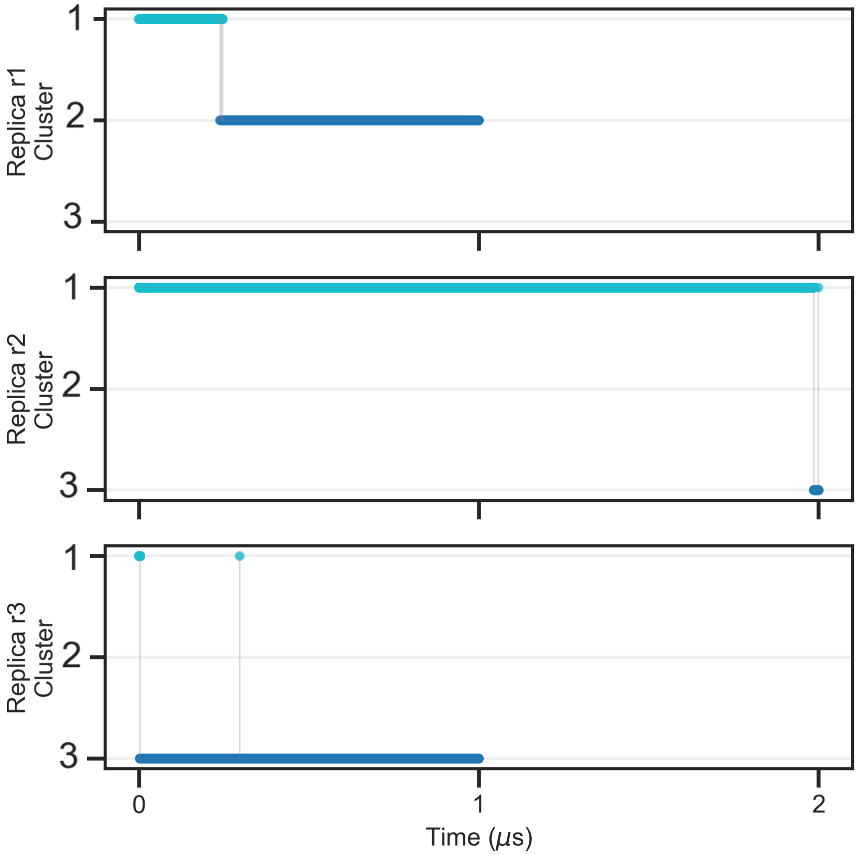

Figure S6: Evolution of **cluster occupancy** during molecular dynamics trajectories. Distribution of structures assigned to clusters CL1, CL2, and CL3 over time for replicas r1–r3. Each color/track represents the structural transition between clusters throughout the classical molecular dynamics runs.

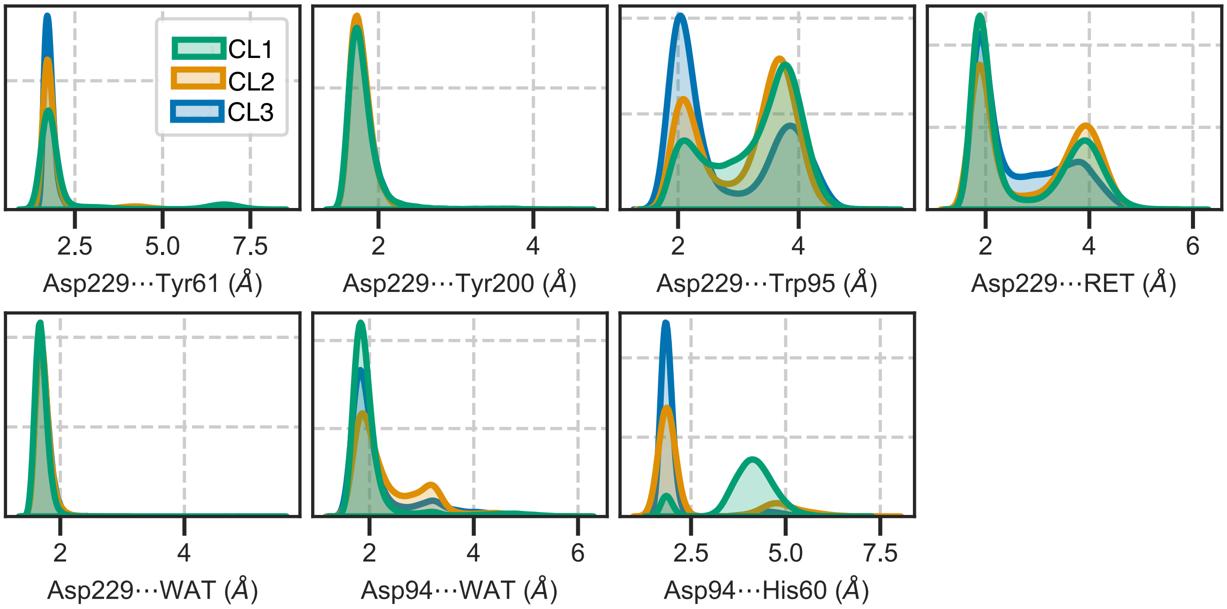

Figure S7: Distribution of **inter-residue distances** within the retinal counterion complex. Each panel shows the distance populations for specific residue pairs, categorized by cluster. These distributions highlight the structural variability and stability of the counterion environment across different conformational states.

S1.4 QM/MM(Pol) DFT calculations

To calculate site energies and couplings of the retinal-salinixanthin (SXN) exciton system, we employ the same QM/MMPol protocol introduced and validated by us in Ref. 27 for Kin4B8-lutein and -zeaxanthin.

Exciton Hamiltonians are constructed using an ensemble of 110 representative structures of Kin4B8–SXN rhodopsin, extracted from set-cis(-) (80 structures) and set-cis(+) (30 structures).

Starting from these configurations, ground-state (S_0) geometry optimizations of the retinal protonated Schiff base (rPSB) and the SXN are carried out, embedding the pigments in the protein+membrane. An electrostatic embedding QM/MM framework is employed. The QM region, comprising both rPSB and SXN (225 atoms), is described at the DFT level using the CAM-B3LYP functional with the 6-31G(d) basis set, including Grimme's D3 dispersion correction with Becke–Johnson damping. The MM region is treated using the same force

field adopted in the MD simulations (see Section S1.2). Amino acid residues (side chains) and water molecules within 4.0 Å of the QM subsystem are allowed to relax, while the remaining part of the system is kept frozen. The total charge of the QM region is set to +1, corresponding to the protonated Schiff base. A hydrogen link atom is introduced at the QM/MM boundary between the C_α and C_β atoms of rPSB.

Site energies We calculate the site energies for both rPSB (S_1) and SXN (S_2) using a polarizable embedding QM/MM (QM/MMPol) approach based on the induced point-dipole formulation.[28–33] We repeat calculations for each pigment, including the other pigment in the QM part, while the other pigment and the rest of the system atoms are put in the MMPol part. MM atoms are represented by fixed point charges and isotropic atomic polarizabilities, allowing the environment to respond self-consistently to the electric field generated by the QM subsystem. The MMPol region is described using the AMBER ffpol12 force field,[34] together with the AL set of atomic polarizabilities reported by Wang *et al.*[35]

To calculate the set of polarizable charges and induced dipoles of the pigment in the MM-Pol part, we calculate polarization-consistent electrostatic potential (ESP) charges. These charges are computed for both rPSB (in Ref. 36) and SXN (in this work) at the B3LYP/6-311G(d,p) level using the Polchat tool.[37] The QM part is described at the QM/MMPol TD-DFT level using the CAM-B3LYP functional and the 6-31+G(d) basis set, while the environment is treated classically as a collection of point charges and induced dipoles.

Excitonic couplings We evaluate the excitonic couplings between rPSB S_1 and Car S_2 by direct integration of transition densities obtained from QM/MMPol calculations.[29,38] The SXN-rPSB coupling is computed as

$$V_{r\text{PSB-SXN}} = \int d\mathbf{r}_1 \int d\mathbf{r}_2 \rho^{tr}_{\text{SXN}}(\mathbf{r}_1) \frac{1}{|\mathbf{r}_1 - \mathbf{r}_2|} \rho^{tr}_{r\text{PSB}}(\mathbf{r}_2) \tag{S1}$$

where ρ_i^{tr} and ρ_j^{tr} denote the corresponding transition densities. In addition, the explicit contribution arising from the polarizable environment is included as

$$V_{r\text{PSB-SXN}}^{MMPol} = -\sum_l \int \mathrm{d}\mathbf{r}' \rho_{\text{SXN}}^{T*}(\mathbf{r}') \cdot \mu_l^{MMPol}(\rho_{r\text{PSB}}^T) \tag{S2}$$

which accounts for the interaction between the transition density of pigment i and the dipoles induced at each polarizable MM site l by the transition density of pigment j.

Dexter exchange contributions are neglected, as they have been shown to be negligible for systems with comparable inter-pigment center-to-center distances (12–13 Å).[39,40] All calculations are performed using a locally modified version of the Gaussian 16 package.[21] Excitonic interactions are analyzed by constructing a two-state (2×2) excitonic Hamiltonian using the EXcitonic Analysis Tool (EXAT).[41]

S1.5 Spectral density calculations

Spectral densities for the bright S_2 state of SXN are obtained from normal-mode analyses[42] performed on ground-state optimized geometries. Huang–Rhys factors and spectral densities are then computed using the formalism described in Section S2.2. Each vibrational mode is broadened using a Lorentzian line shape with a half-width at half-maximum of 5 cm^{-1}.

To improve the statistical description of the spectral densities, results are averaged over 10 representative Kin4B8–salinixanthin structures sampled from both set-cis(-) and set-cis(+). For each structure, frequency and excited-state gradient calculations are carried out on the S_0 QM/MM-optimized geometries described in Section S1.4. The spectral density of retinal, computed in Kin4B8 protein environment and using the same methodology, is taken from Ref. 27, as it is found to be nearly identical across all Kin4B8–Car complexes.

Starting from CAM-B3LYP/6-31G(d) optimized geometries, vibrational frequencies are computed at the B3LYP/6-311+G(d,p) level of theory with the MM region fully frozen. Excited-

state gradients are obtained from TD-DFT calculations performed at the same level of theory. All QM/MM calculations are carried out using the Gaussian 16 software package.[21]

Coupling between the electronic excitations and the protein environment is modeled by adding a low-frequency contribution to the spectral density, described by an underdamped Brownian oscillator with reorganization energy $\lambda_{\mathrm{env}} = 37$ cm^{-1} and damping constant $\gamma_{\mathrm{env}} = 30$ cm^{-1}.[43] The resulting total (vibrational + environmental) spectral densities are shown in Figure S8a,b.

To assess the effect of the protein environment on the SXN spectral density in the Kin4B8 complex, we removed the environment and repeated the geometry optimization, frequency, and gradient calculations on the isolated SXN chromophore. This analysis was performed for a representative frame extracted from the set-*cis*(−) ensemble. The resulting spectral density (Figure S8c) yields a total reorganization energy of 2747 cm^{-1}.

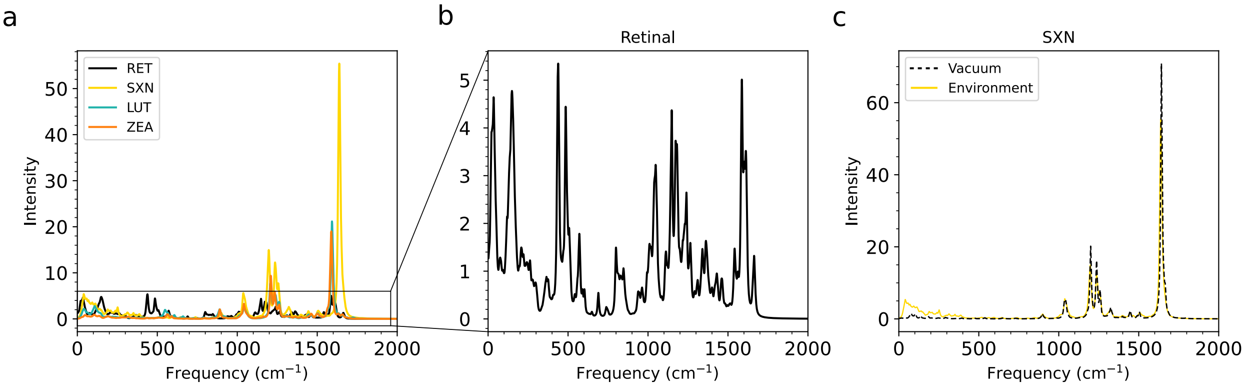

Figure S8: Total (vibrational + environmental) **spectral densities** (SDs). All curves are reported as $J(\omega)/\omega$ (dimensionless) to facilitate comparison in the low-frequency region. **a**: Spectral densities are shown for each of the three carotenoids (SXN, LUT, and ZEA) computed within their respective Kin4B8–Car complexes. The retinal SD is obtained as the average of the retinal SDs in the Kin4B8–ZEA and Kin4B8–LUT complexes. **b**: inset highlighting the retinal SD. **c**: spectral density of salinaxanthin computed in the Kin4B8 environment and in vacuum.

S2 Theory

S2.1 Exciton Hamiltonian

The electronic Hamiltonian of the salinixanthin–retinal dimer is described within the Frenkel exciton framework as:

$$\hat{\mathcal{H}}_{\text{exc}} = \sum_i \mathcal{E}_i |i\rangle \langle i| + \sum_{i \neq j} V_{ij} |i\rangle \langle j| , \tag{S3}$$

where $|i\rangle$ denotes the excited state localized on pigment i, characterized by a site energy $\mathcal{E}_i$, and V_{ij} represents the excitonic coupling between pigments i and j.

The eigenstates of $\hat{\mathcal{H}}_{\text{exc}}$ define the excitons:

$$|a\rangle = \sum_i c_{ia} |i\rangle , \tag{S4}$$

where the coefficients c_{ia} form the eigenvector matrix $\mathbf{c}$ of $\mathbf{H}_{\text{exc}}$. The corresponding eigenvalues E_a give the exciton energies. Throughout this work, indices a, b refer to excitonic states, while i, j label localized excitations.

S2.2 Coupling to vibrations

In our vibronic model, the electronic couplings are kept fixed, whereas the site energies fluctuate due to the nuclear motion. Both ground and excited electronic states are represented by harmonic potential energy surfaces sharing the same curvature, defined by the ground-state normal modes with frequencies ω_k. The excited-state minima are displaced relative to the ground state along these modes, and the magnitude of each displacement is encoded in the Huang–Rhys factor S_k. These factors are obtained by projecting the excited-state vertical gradient[38,42] f_k onto the ground-state normal modes:

$$S_k = \frac{f_k^2}{2\omega_k^3}. \tag{S5}$$

The vibronic coupling is incorporated through the spectral density,

$$J_i(\omega) = \pi \sum_k \omega_k \lambda_{k,i}\, \delta(\omega - \omega_{k,i}),$$

where $\lambda_{k,i} = S_{k,i}\omega_{k,i}$ is the contribution of mode k to the reorganization energy of pigment i. The total reorganization energy of pigment i is then:

$$\lambda_i = \frac{\hbar}{\pi} \int_0^{\infty} \mathrm{d}\omega\, \frac{J_i(\omega)}{\omega} = \sum_k \lambda_{k,i}. \tag{S6}$$

In this work, correlations between fluctuations on different pigments are neglected, so to each pigment is assigned an independent spectral density.

The spectral density determines the bath correlation function,

$$C_i(t) = \frac{1}{\pi} \int_0^{\infty} \mathrm{d}\omega \left[\coth\!\left(\frac{\beta\hbar\omega}{2}\right) \cos(\omega t) - i \sin(\omega t)\right] J_i(\omega), \tag{S7}$$

which enters the expressions for spectroscopy and energy-transfer rates.

S2.3 Spectroscopy

Absorption spectra are computed using the full cumulant expansion formalism, where the dipole autocorrelation function is expanded to second order. The resulting absorption lineshape is given by:[44]

$$A(\omega) \propto \omega\, \Re \int_{-\infty}^{+\infty} \mathrm{d}t\, e^{-i\omega t} \sum_{ab} \mathbf{I}_{ab}(t)\, \mathbf{M}_{ab}, \tag{S8}$$

where $\mathbf{M}_{ab} = \vec{\mu}_a \cdot \vec{\mu}_b$ is the dipole strength matrix in the exciton basis, and the excitonic transition dipoles are obtained as $\vec{\mu}_a = \sum_i \vec{\mu}_i c_{ia}$.

The absorption tensor is

$$\mathbf{I}(t) = e^{-i\mathbf{H}_{\mathrm{exc}} t}\, e^{-\mathbf{K}(t)}, \tag{S9}$$

where $\mathbf{H}_{\text{exc}}$ is diagonal in the exciton basis, with diagonal elements being E_a, and $\mathbf{K}(t)$ is the lineshape matrix:

$$K_{ab} = \sum_c \sum_i c_{ia}^* |c_{ic}|^2 c_{ib} \int_0^t \mathrm{d}t_2 \int_0^{t_2} \mathrm{d}t_1\, e^{i\omega_{ac}t_2 - i\omega_{bc}t_1} C_i(t_1). \tag{S10}$$

Here, $\hbar\omega_{ac} = E_a - E_c$ is the exciton energy gap.

CD spectra are obtained by replacing the dipole strength matrix with the rotatory strength matrix $\mathbf{r}$. Following Ref. 41, but performing the geometric averaging $\sqrt{\mathcal{E}_i\mathcal{E}_j}$ in the site basis as in Ref. 45, and symmetrizing the final expression, we use:

$$r_{ab} = -\frac{e\hbar}{4\pi m_e} \sum_{ij} c_{ia} c_{jb} \frac{\vec{\nabla}_i \cdot \vec{m}_j + \vec{\nabla}_j \cdot \vec{m}_i}{\sqrt{\mathcal{E}_i \mathcal{E}_j}}, \tag{S11}$$

where $\vec{\nabla}$ is the electric dipole operator in the velocity gauge and $\vec{m}$ is the magnetic dipole moment.

Absorption and CD spectra are computed for each snapshot s and then ensemble-averaged within each set, as:

$$\langle A(\omega) \rangle_{\text{set}} = \frac{1}{N_{\text{set}}} \sum_{s \in \text{set}} A_s(\omega)$$

this accounts for the inhomogeneous broadening arising from multiple pigment-environment configurations.

S2.4 EET rates and kinetic model

Excitation-energy transfer is described using a Markovian kinetic scheme based on Förster theory. The transfer rate from donor D to acceptor A is:[38,46,47]

$$k_{DA} = 2\frac{|V_{DA}|^2}{\hbar^2} J_{DA}, \tag{S12}$$

$$J_{DA} = \int_0^{+\infty} \mathrm{d}\omega \, \tilde{A}_A(\omega) \, \tilde{F}_D(\omega), \tag{S13}$$

where V_{DA} is the excitonic coupling and J_{DA} the spectral overlap. $\tilde{A}_A(\omega)$ and $\tilde{F}_D(\omega)$ are the area-normalized absorption $A_A(\omega)$ and fluorescence $F_D(\omega)$ lineshapes of D and A. These are computed as:[48,49]

$$A_A(\omega) = \Re \int_0^{\infty} \mathrm{d}t \, e^{-i(\omega_A - \omega)t - g_A(t)}, \tag{S14}$$

$$F_D(\omega) = \Re \int_0^{\infty} \mathrm{d}t \, e^{-i(\omega_D - \omega + 2\lambda_D)t - g_D^*(t)}, \tag{S15}$$

where λ_D is the reorganization energy and the lineshape function is

$$g_A(t) = \int_0^{t} \mathrm{d}t_2 \int_0^{t_2} \mathrm{d}t_1 \, C_A(t_1). \tag{S16}$$

The area-normalization is:

$$\tilde{A}(\omega) = \frac{A(\omega)}{\int_0^{\infty} \mathrm{d}\omega A(\omega)} \tag{S17}$$

and similarly for $F\tilde{(\omega)}$.

The excited-state electronic populations evolve according to the Pauli master equation:

$$\frac{\mathrm{d}P_i(t)}{\mathrm{d}t} = \sum_{j \neq i} \Big(k_{j \to i} P_j(t) - k_{i \to j} P_i(t) \Big) \tag{S18}$$

where $i, j \in \{S_2^{\mathrm{car}}, S_1^{r\mathrm{PSB}}, \mathrm{GS}\}$ and we drop any distinction between the carotenoid's S_1 state and its GS as we assume that the S_1 state relaxes directly into the GS. The forward and backwards EET rates are computed as explained above, while the internal conversion rate $k_{\mathrm{IC}} = k_{S_2^{\mathrm{car}} \to \mathrm{GS}}$ is the inverse of the carotenoid lifetime in absence of EET (τ_D).

The energy transfer efficiency can be estimated as

$$\Phi = \frac{k_{\mathrm{EET}}}{k_{\mathrm{EET}} + k_{\mathrm{IC}}} = \frac{\tau_D}{\tau_{\mathrm{EET}} + \tau_D} \tag{S19}$$

where $k_{\mathrm{EET}} \equiv k_{S_2^{\mathrm{car}} \to S_1^{\mathrm{car}}}$ and $\tau_{\mathrm{EET}} = 1/k_{\mathrm{EET}}$. This efficiency is then multiplied by 100 to transform to percentage.

Throughout this paper we report the average EET time, rate and average efficiency calculated as follows for each set of snapshots:

$$\langle \tau_{\mathrm{EET}} \rangle_{\mathrm{set}} = \frac{1}{N_{\mathrm{set}}} \sum_{s \in \mathrm{set}} \tau_{\mathrm{EET}}^{(s)},$$

$$\langle k_{\mathrm{EET}} \rangle_{\mathrm{set}} = \frac{1}{N_{\mathrm{set}}} \sum_{s \in \mathrm{set}} k_{\mathrm{EET}}^{(s)},$$

$$\langle \Phi \rangle_{\mathrm{set}} = \frac{1}{N_{\mathrm{set}}} \sum_{s \in \mathrm{set}} \Phi_s,$$

where Φ_s is the EET efficiency calculated for snapshot s with $k_{\mathrm{EET}}^{(s)}$.

S3 Color tuning analysis

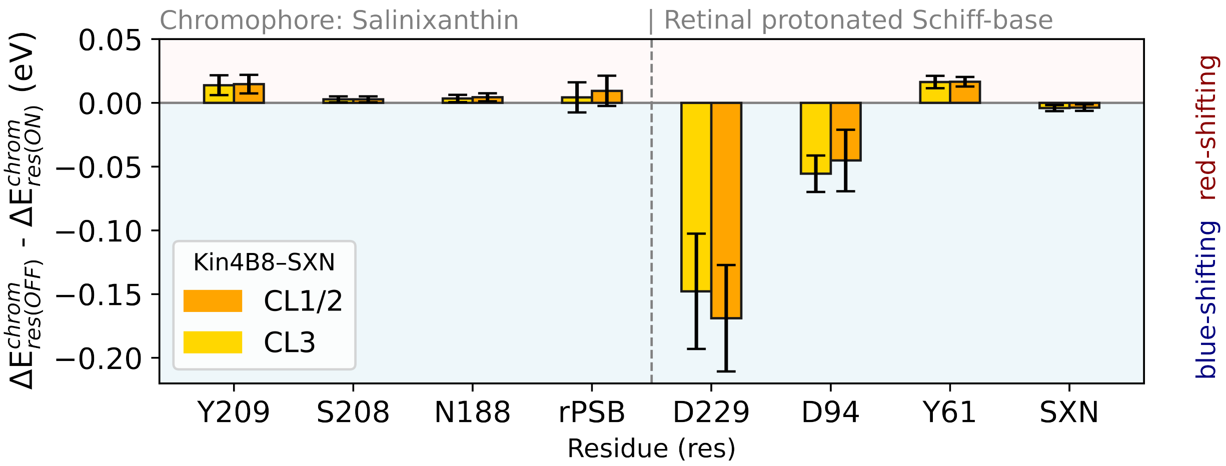

Figure S9: **Color-tuning analysis** for salinixanthin and rPSB in the Kin4B8–SXN complex. Average change in chromophore excitation energies, $\Delta E^{\mathrm{chrom}}\mathrm{res(OFF)} - \Delta E^{\mathrm{chrom}}\mathrm{res(ON)}$ (eV), upon electrostatic turn-off of selected residues. Error bars represent the standard deviation over the sampled structures. Blue and red shaded regions indicate blue- and red-shift contributions, respectively. For retinal, the obtained trends are consistent with those reported for the Kin4B8–ZEA and Kin4B8–LUT complexes in Ref. 27, indicating that the identity of the carotenoid does not significantly affect the electrostatic environment of rPSB.

S4 Decomposition of absorption and circular dichroism spectra

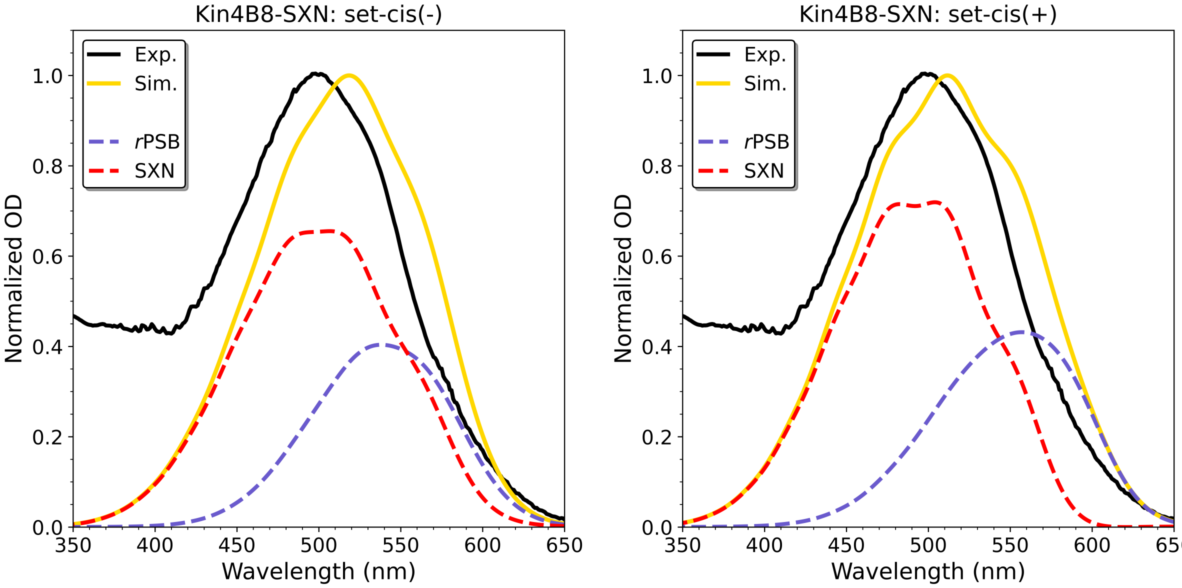

Figure S10: **Simulated absorption spectra** for set-cis(-) (left) and set-cis(+) (right) of the Kin4B8–ZEA complex. Each spectrum represents the ensemble average over $N = 79$ and 30 replicas, respectively. Contributions from individual chromophores are shown. Experimental spectra are adapted with permission from Ref. 1. The experimental and calculated spectra are normalized separately to the central carotenoid peak.

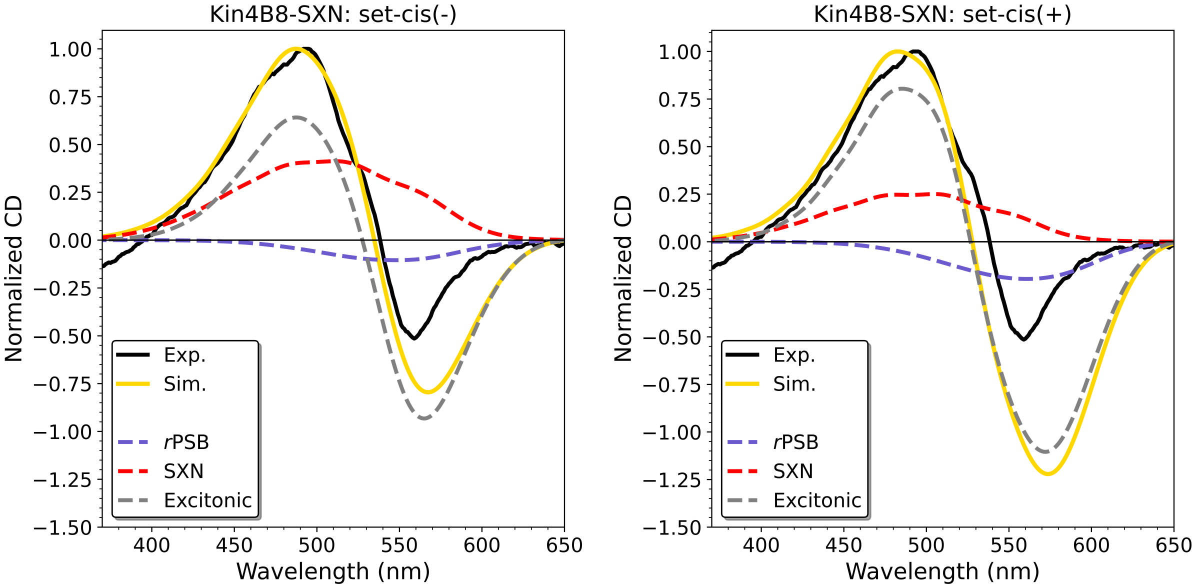

Figure S11: **Simulated circular dichroism spectra** for set-cis(-) (left) and set-cis(+) (right) of the Kin4B8–ZEA complex. Each spectrum represents the ensemble average over $N = 79$ and 30 replicas, respectively. Contributions from individual chromophores are shown, along with the excitonic contribution in the CD spectra. Experimental spectra are adapted with permission from Ref. 1. The experimental and calculated spectra are normalized separately to the highest positive peak.

S5 Effect of salinixanthin excitation energy and its vibronic reorganization energy on EET

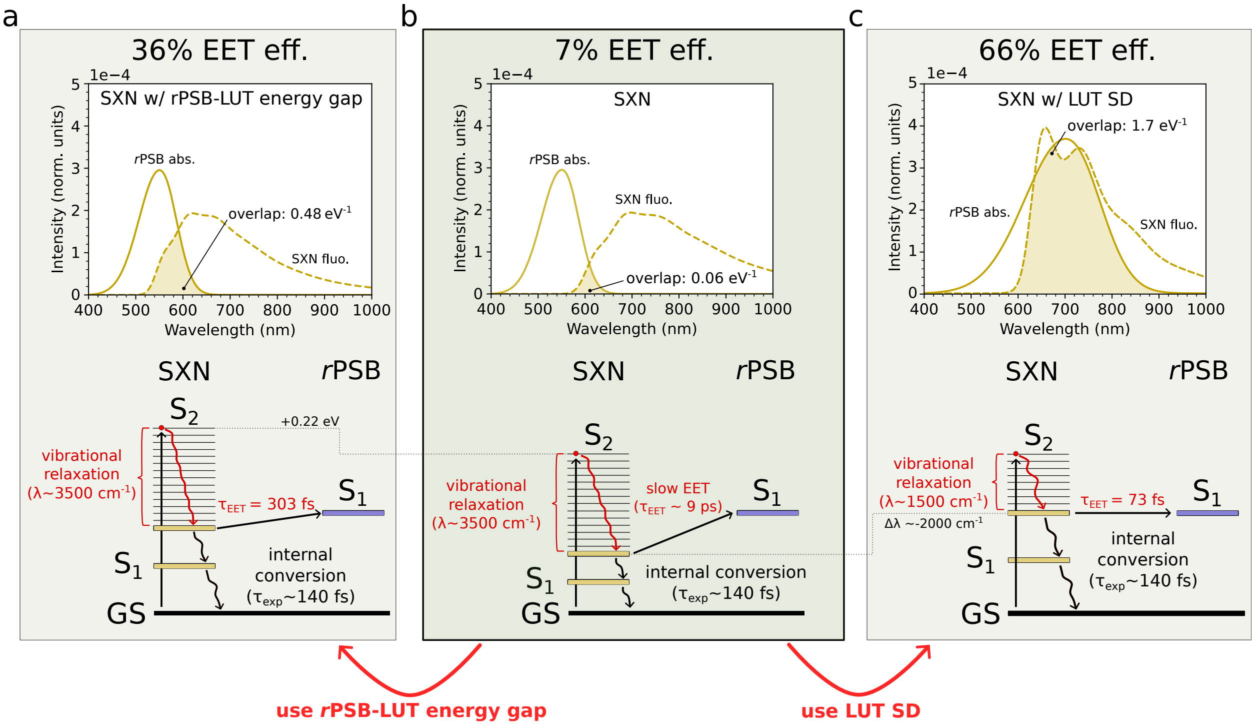

Figure S12: **Spectral-overlap analysis** for salinixanthin (SXN) and two artificial SXN models designed to disentangle the roles of the donor–acceptor energy gap and the carotenoid reorganization energy. **Upper panels**: computed spectral overlap between the calculated SXN fluorescence (fluo.) and the calculated *r*PSB absorption (abs.) for the three models. **Lower panels**: vibronic Jablonski diagrams illustrating the ground state, the carotenoid S_2 state, and the *r*PSB S_1 state, together with the corresponding vibronic relaxation on the S_2 manifold and the associated reorganization energies. Reported times refer to carotenoid internal conversion to the ground state and to Car → *r*PSB excitation energy transfer (EET); for consistency, the same experimental ground-state relaxation time used for LUT-binding Kin4B8 (τ = 140 fs)[1] is adopted here. **(a)** SXN with the CAR-*r*PSB energy gap determined for the lutein(LUT)-Kin4B8 complex: the SXN site energies is blue-shifted by 0.22 eV. **(b)** Original SXN model ($\lambda_{\text{reorg}}^{SXN}$=3593 cm^{-1}). **(c)** SXN with the LUT spectral density ($\lambda_{\text{reorg}}^{LUT}$=1787 cm^{-1}).

In the main text, we have shown that salinixanthin exhibits markedly lower energy-transfer efficiency than lutein (LUT) and zeaxanthin (ZEA). While exciton couplings are observed to be similar among the different complexes, both vibronic and exciton features of SXN distinguish it from the hydroxylated xanthophylls: in the SXN complex, we observed a sub-

stantially larger reorganization energy on the S_2 state and a smaller donor–acceptor energy gap. Both effects reduce the spectral overlap with the retinal S_1 transition, and both could in principle contribute to the inefficient EET observed experimentally. To determine whether these two factors play comparable roles or whether one of them dominates, we constructed two artificial Kin4B8-SXN models: one where the energy gap is selectively modified and the other where the spectral density is modified, while keeping all other parameters unchanged. For reference, panel (b) of Figure S12 reports the unmodified SXN model, which displays the characteristic broad emission profile and minimal overlap with the retinal acceptor.

Effect of the energy gap. In the first model, shown in Figure S12a, the SXN S_2 site energy is artificially blue-shifted by +0.22 eV so as to reproduce the retinal–carotenoid energy gap characteristic of the efficient Kin4B8–LUT complex (0.43 eV). Importantly, the SXN spectral density, and in turn its large reorganization energy of 3593 cm^{-1}, is left untouched. The resulting spectral overlap increases from the original value of 0.06 eV^{-1} to 0.48 eV^{-1}. Although this represents a clear improvement, the overlap remains far below the ∼1.5 eV^{-1} observed with ZEA and LUT, and the corresponding EET time (303 fs) is still an order of magnitude slower when compared to LUT (60- fs). This indicates that restoring a favorable energy gap alone is not sufficient to recover fast transfer.

Effect of the reorganization energy. The second model, shown in Figure S12c, keeps the SXN site energies at their original values but replaces the SXN spectral density with that of LUT which corresponds to a reorganization energy of ∼1800 cm^{-1}. This modification dramatically blue-shifts (and also narrows) the SXN emission, making both the spectral overlap (1.7 eV^{-1}) and the predicted EET time (73 fs) comparable to that of ZEA and LUT. Notably, this recovery occurs despite the fact that the SXN S_2 energy remains red-shifted relative to the hydroxylated xanthophylls.

Taken together, these artificial-models demonstrate that the reduced rPSB-SXN energy gap

plays only a secondary role, while it is the large reorganization energy that is the predominant factor suppressing spectral overlap and limiting EET efficiency in the Kin4B8–SXN complex.

S6 Population dynamics

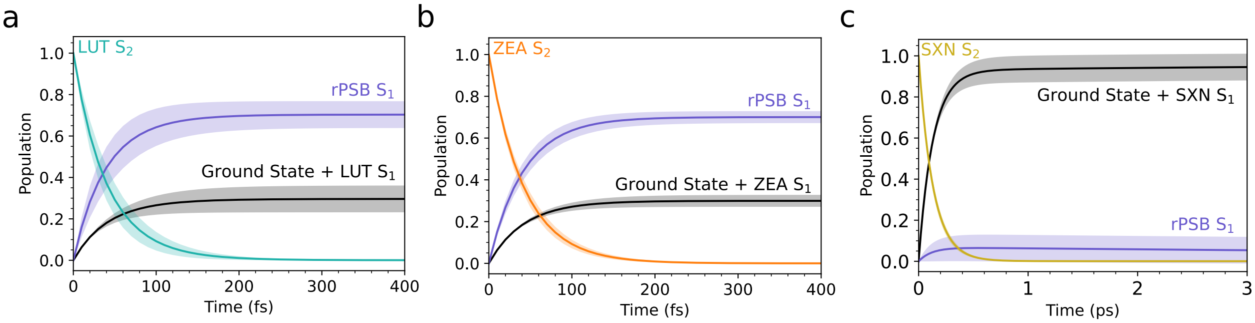

Figure S13: **Population dynamics in different caroteoinds (Cars)-Kin4B8 complex**. (a) lutein (LUT)-Kin4B8, (b) zeaxanthin (ZEA)-Kin4B8 and (c) salinixanthin (SXN)-Kin4B8. The temperature is set to 298 K. The initial population is set on the Car S_2 state, and then transferred to the rPSB S_1 state (blue) and to an artificial state colored in black which represent the sum of the S_1 Car + ground state. This state isconnected to the S_2 Car state through a τ_{GS}=140 fs[1] downhill-only rate. The error bands show the standard deviation of the populations associated to the average. For SXN, we average over set-cis(-) of MD simulations.

References

(1) Chazan, A.; Das, I.; Fujiwara, T.; Murakoshi, S.; Rozenberg, A.; Molina-Márquez, A.; Sano, F. K.; Tanaka, T.; Gómez-Villegas, P.; Larom, S.; Pushkarev, A.; Malakar, P.; Hasegawa, M.; Tsukamoto, Y.; Ishizuka, T.; Konno, M.; Nagata, T.; Mizuno, Y.; Katayama, K.; Abe-Yoshizumi, R.; Ruhman, S.; Inoue, K.; Kandori, H.; León, R.; Shihoya, W.; Yoshizawa, S.; Sheves, M.; Nureki, O.; Béjà, O. Phototrophy by Antenna-Containing Rhodopsin Pumps in Aquatic Environments. *Nature* **2023**, *615*, 535–540.

(2) Pedraza-González, L.; De Vico, L.; Marín, M. d. C.; Fanelli, F.; Olivucci, M. a-ARM: Automatic Rhodopsin Modeling with Chromophore Cavity Generation, Ionization State Selection and External Counter-ion Placement. *J. Chem. Theory Comput.* **2019**, *15*, 3134–3152.

(3) Maier, J. A.; Martinez, C.; Kasavajhala, K.; Wickstrom, L.; Hauser, K. E.; Simmerling, C. ff14SB: Improving the Accuracy of Protein Side Chain and Backbone Parameters from ff99SB. *J. Chem. Theory Comput.* **2015**, *11*, 3696–3713.

(4) Morris, G. M.; Huey, R.; Lindstrom, W.; Sanner, M. F.; Belew, R. K.; Goodsell, D. S.; Olson, A. J. AutoDock4 and AutoDockTools4: Automated Docking with Selective Receptor Flexibility. *J. Comput. Chem.* **2009**, *30*, 2785–2791.

(5) Luecke, H.; Schobert, B.; Stagno, J.; Imasheva, E. S.; Wang, J. M.; Balashov, S. P.; Lanyi, J. K. Crystallographic structure of xanthorhodopsin, the light-driven proton pump with a dual chromophore. *Proc. Natl. Acad. Sci. USA* **2008**, *105*, 16561–16565.

(6) Jo, S.; Kim, T.; Iyer, V. G.; Im, W. CHARMM-GUI: A web-based graphical user interface for CHARMM. *J. Comput. Chem.* **2008**, *29*, 1859–1865.

(7) Jorgensen, W. L.; Chandrasekhar, J.; Madura, J. D.; Impey, R. W.; Klein, M. L. Comparison of simple potential functions for simulating liquid water. *J. Chem. Phys.* **1983**, *79*, 926–935.

(8) Dickson, C. J.; Walker, R. C.; Gould, I. R. Lipid21: Complex Lipid Membrane Simulations with AMBER. *J. Chem. Theory Comput.* **2022**, *18*, 1726–1736.

(9) Melaccio, F.; del Carmen Marín, M.; Valentini, A.; Montisci, F.; Rinaldi, S.; Cherubini, M.; Yang, X.; Kato, Y.; Stenrup, M.; Orozco-González, Y.; Ferre, N.; Luk, H. L.; Kandori, H.; Olivucci, M. Toward Automatic Rhodopsin Modeling as a Tool for High-Throughput Computational Photobiology. *JCTC* **2016**, *12*, 6020–6034.

(10) Prandi, I. G.; Viani, L.; Andreussi, O.; Mennucci, B. Combining Classical Molecular Dynamics and Quantum Mechanical Methods for the Description of Electronic Excitations: The Case of Carotenoids. *J. Comput. Chem.* **2016**, *37*, 981–991.

(11) Wang, J.; Wolf, R. M.; Caldwell, J. W.; Kollman, P. A.; Case, D. A. Development and testing of a general amber force field. *J. Comput. Chem.* **2004**, *25*, 1157–1174.

(12) Bayly, C. I.; Cieplak, P.; Cornell, W. D.; Kollman, P. A. A Well-Behaved Electrostatic Potential Based Method Using Charge Restraints for Deriving Atomic Charges: The RESP Model. *J. Phys. Chem.* **1993**, *97*, 10269–10280.

(13) Lee, C.; Yang, W.; Parr, R. G. Development of the Colle-Salvetti correlation-energy formula into a functional of the electron density. *Phys. Rev. B.* **1988**, *37*, 785.

(14) Becke, A. D. Density-functional thermochemistry. III. The role of exact exchange. *J. Chem. Phys.* **1993**, *98*, 5648–5652.

(15) Cancès, E.; Mennucci, B.; Tomasi, J. A new integral equation formalism for the polarizable continuum model: Theoretical background and applications to isotropic and anisotropic dielectrics. *J. Chem. Phys.* **1997**, *107*, 3032–3041.

(16) Tomasi, J.; Mennucci, B.; Cammi, R. Quantum Mechanical Continuum Solvation Models. *Chem. Rev.* **2005**, *105*, 2999–3094.

(17) Scalmani, G.; Frisch, M. J. Continuous surface charge polarizable continuum models of solvation. I. General formalism. *J. Chem. Phys.* **2010**, *132*, 114110.

(18) Seabra, G. M.; Swails, J.; Roitberg, A. E. *Multi-scale Quantum Models for Biocatalysis*; Springer Netherlands, 2009; p 3–20.

(19) Walker, R. C.; Crowley, M. F.; Case, D. A. The implementation of a fast and accurate QM/MM potential method in Amber. *J. Comput. Chem.* **2008**, *29*, 1019–1031.

(20) Zhao, S.; Wei, H.; Cieplak, P.; Duan, Y.; Luo, R. PyRESP: A Program for Electrostatic Parameterizations of Additive and Induced Dipole Polarizable Force Fields. *J. Chem. Theory Comput.* **2022**, *18*, 3654–3670.

(21) Frisch, M. J.; Trucks, G. W.; Schlegel, H. B.; Scuseria, G. E.; Robb, M. A.; Cheeseman, J. R.; Scalmani, G.; Barone, V.; Petersson, G. A.; Nakatsuji, H.; Li, X.; Caricato, M.; Marenich, A. V.; Bloino, J.; Janesko, B. G.; Gomperts, R.; Mennucci, B.; Hratchian, H. P.; Ortiz, J. V.; Izmaylov, A. F.; Sonnenberg, J. L.; Williams-Young, D.; Ding, F.; Lipparini, F.; Egidi, F.; Goings, J.; Peng, B.; Petrone, A.; Henderson, T.; Ranasinghe, D.; Zakrzewski, V. G.; Gao, J.; Rega, N.; Zheng, G.; Liang, W.; Hada, M.; Ehara, M.; Toyota, K.; Fukuda, R.; Hasegawa, J.; Ishida, M.; Nakajima, T.; Honda, Y.; Kitao, O.; Nakai, H.; Vreven, T.; Throssell, K.; Montgomery, J. A., Jr.; Peralta, J. E.; Ogliaro, F.; Bearpark, M. J.; Heyd, J. J.; Brothers, E. N.; Kudin, K. N.; Staroverov, V. N.; Keith, T. A.; Kobayashi, R.; Normand, J.; Raghavachari, K.; Rendell, A. P.; Burant, J. C.; Iyengar, S. S.; Tomasi, J.; Cossi, M.; Millam, J. M.; Klene, M.; Adamo, C.; Cammi, R.; Ochterski, J. W.; Martin, R. L.; Morokuma, K.; Farkas, O.; Foresman, J. B.; Fox, D. J. Gaussian 16 Revision A.03. 2016; Gaussian Inc. Wallingford CT.

(22) Case, D. A.; Aktulga, H. M.; Belfon, K.; Ben-Shalom, I. Y.; Berryman, J. T.; Brozell, S. R.; Cerutti, D. S.; Cheatham III, T. E.; Cruzeiro, V. W. D.; Darden, T. A.;

Duke, R. E.; Giambasu, G.; Gilson, M. K.; Gohlke, H.; Goetz, A. W.; Harris, R.; Izadi, S.; Izmailov, S. A.; Jin, C.; Kasavajhala, K.; Kaymak, M. C.; King, E.; Kovalenko, A.; Kurtzman, T.; Lee, T. S.; LeGrand, S.; Li, P.; Lin, C.; Liu, J.; Luchko, T.; Luo, R.; Machado, M.; Man, V.; Manathunga, M.; Merz, K. M.; Miao, Y.; Mikhailovskii, O.; Monard, G.; Nguyen, H.; O'Hearn, K. A.; Onufriev, A.; Pan, F.; Pantano, S.; Qi, R.; Rahnamoun, A.; Roe, D. R.; Roitberg, A.; Sagui, C.; Schott-Verdugo, S.; Shajan, A.; Simmerling, C. L.; Skrynnikov, N. R.; Smith, J. C.; Swails, J.; Walker, R. C.; Wang, J.; Wei, H.; Wolf, R.; Wu, X.; Xiong, Y.; Xue, Y.; York, D. M.; Zhao, S.; Kollman, P. A. Amber 2022. 2022; University of California, San Francisco.

(23) Salomon-Ferrer, R.; Götz, A. W.; Poole, D.; Le Grand, S.; Walker, R. C. Routine Microsecond Molecular Dynamics Simulations with AMBER on GPUs. 2. Explicit Solvent Particle Mesh Ewald. *J. Chem. Theory Comput.* **2013**, *9*, 3878–3888.

(24) Götz, A. W.; Williamson, M. J.; Xu, D.; Poole, D.; Le Grand, S.; Walker, R. C. Routine Microsecond Molecular Dynamics Simulations with AMBER on GPUs. 1. Generalized Born. *J. Chem. Theory Comput.* **2012**, *8*, 1542–1555.

(25) Le Grand, S.; Götz, A. W.; Walker, R. C. SPFP: Speed without Compromise—A Mixed Precision Model for GPU Accelerated Molecular Dynamics Simulations. *Comput. Phys. Commun.* **2013**, *184*, 374–380.

(26) McInnes, L.; Healy, J.; Melville, J. UMAP: Uniform Manifold Approximation and Projection for Dimension Reduction. **2018**,

(27) Salvadori, G.; Saraceno, P.; Santomieri, A.; John, C.; Pedraza-González, L. Structural and spectroscopic basis of excitation energy transfer in microbial rhodopsins binding xanthophylls. *Chem. Sci.* **2025**, *16*, 18423–18437.

(28) Bondanza, M.; Nottoli, M.; Cupellini, L.; Lipparini, F.; Mennucci, B. Polarizable Em-

bedding QM/MM: The Future Gold Standard for Complex (Bio)Systems? *Phys. Chem. Chem. Phys.* **2020**, *22*, 14433–14448.

(29) Curutchet, C.; Muñoz-Losa, A.; Monti, S.; Kongsted, J.; Scholes, G. D.; Mennucci, B. Electronic Energy Transfer in Condensed Phase Studied by a Polarizable QM/MM Model. *J. Chem. Theory Comput.* **2009**, *5*, 1838–1848.

(30) Lipparini, F. General Linear Scaling Implementation of Polarizable Embedding Schemes. *J. Chem. Theory Comput.* **2019**, *15*, 4312–4317.

(31) Loco, D.; Lagardère, L.; Adjoua, O.; Piquemal, J.-P. Atomistic Polarizable Embeddings: Energy, Dynamics, Spectroscopy, and Reactivity. *Acc. Chem. Res.* **2021**, *54*, 2812–2822.

(32) Nottoli, M.; Bondanza, M.; Mazzeo, P.; Cupellini, L.; Curutchet, C.; Loco, D.; Lagardère, L.; Piquemal, J.-P.; Mennucci, B.; Lipparini, F. QM/AMOEBA Description of Properties and Dynamics of Embedded Molecules. *WIREs Comput. Mol. Sci.* **2023**, *13*, e1674.

(33) Cupellini, L.; Caprasecca, S.; Guido, C. A.; Müh, F.; Renger, T.; Mennucci, B. Coupling to Charge Transfer States is the Key to Modulate the Optical Bands for Efficient Light Harvesting in Purple Bacteria. *J. Phys. Chem. Lett.* **2018**, *9*, 6892–6899.

(34) Wang, J.; Cieplak, P.; Li, J.; Cai, Q.; Hsieh, M.-J.; Luo, R.; Duan, Y. Development of Polarizable Models for Molecular Mechanical Calculations. 4. Van Der Waals Parametrization. *J. Phys. Chem. B* **2012**, *116*, 7088–7101.

(35) Wang, J.; Cieplak, P.; Li, J.; Hou, T.; Luo, R.; Duan, Y. Development of Polarizable Models for Molecular Mechanical Calculations I: Parameterization of Atomic Polarizability. *J. Phys. Chem. B* **2011**, *115*, 3091–3099.

(36) Di Prima, D.; Pedraza-González, L.; Reinholdt, P.; Kongsted, J.; Mennucci, B. Flu-

orescent Rhodopsins: A Challenging Test for Cost-Effective QM/MM Approaches. *J. Phys. Chem. A* **2024**, *129*, 1769–1778.

(37) Caprasecca, S.; Curutchet, C.; Mennucci, B.; Jurinovich, S. PolChat: A Polarisation-Consistent Charge Fitting Tool, Molecolab Tools. `https://github.com/Molecolab-Pisa/polchat`, 2017; (accessed 2025-09-05).

(38) Cupellini, L.; Corbella, M.; Mennucci, B.; Curutchet, C. Electronic Energy Transfer in Biomacromolecules. *WIREs Comput. Mol. Sci.* **2019**, *9*, e1392.

(39) Fujimoto, K. J.; Balashov, S. P. Vibronic Coupling Effect on Circular Dichroism Spectrum: Carotenoid–Retinal Interaction in Xanthorhodopsin. *J. Chem. Phys.* **2017**, *146*.

(40) Fujimoto, K. J. Electronic Couplings and Electrostatic Interactions Behind the Light Absorption of Retinal Proteins. *Front. Mol. Biosci.* **2021**, *8*, 752700.

(41) Jurinovich, S.; Cupellini, L.; Guido, C. A.; Mennucci, B. EXAT: EXcitonic Analysis Tool. *J. Comput. Chem.* **2018**, *39*, 279–286.

(42) Lee, M. K.; Huo, P.; Coker, D. F. Semiclassical Path Integral Dynamics: Photosynthetic Energy Transfer with Realistic Environment Interactions. *Annu. Rev. Phys. Chem.* **2016**, *67*, 639–668.

(43) Saraceno, P.; Sláma, V.; Cupellini, L. First-Principles Simulation of Excitation Energy Transfer and Transient Absorption Spectroscopy in the CP29 Light-Harvesting Complex. *J. Chem. Phys.* **2023**, *159*, 184112.

(44) Ma, J.; Cao, J. Förster Resonance Energy Transfer, Absorption and Emission Spectra in Multichromophoric Systems. I. Full Cumulant Expansions and System-Bath Entanglement. *J. Chem. Phys.* **2015**, *142*, 094106.

(45) Cupellini, L.; Lipparini, F.; Cao, J. Absorption and Circular Dichroism Spectra of

Molecular Aggregates with the Full Cumulant Expansion. *J. Phys. Chem. B* **2020**, *124*, 8610–8617.

(46) Förster, T. Energy migration and fluorescence. *J. Biomed. Opt.* **2012**, *17*, 011002.

(47) Yang, M.; Fleming, G. R. Influence of phonons on exciton transfer dynamics: comparison of the Redfield, Förster, and modified Redfield equations. *Chem. Phys.* **2002**, *275*, 355–372.

(48) Nöthling, J. A.; Mančal, T.; Krüger, T. P. J. Accuracy of approximate methods for the calculation of absorption-type linear spectra with a complex system–bath coupling. *J. Chem. Phys* **2022**, *157*, 095103.

(49) Renger, T.; Marcus, R. A. On the relation of protein dynamics and exciton relaxation in pigment–protein complexes: An estimation of the spectral density and a theory for the calculation of optical spectra. *J. Chem. Phys* **2002**, *116*, 9997–10019.
